# Divergence in heritable life history traits suggests potential for local adaptation and trade‐offs associated with a coal ash disposal site

**DOI:** 10.1111/eva.13256

**Published:** 2021-06-09

**Authors:** R. Wesley Flynn, Allison M. Welch, Stacey L. Lance

**Affiliations:** ^1^ Savannah River Ecology Laboratory University of Georgia Aiken SC USA; ^2^ Department of Biology College of Charleston Charleston SC USA

**Keywords:** amphibian, environmental contaminant, in situ, plasticity, quantitative genetics

## Abstract

Globally, human activities have resulted in rapid environmental changes that present unique challenges for wildlife. However, investigations of local adaptation in response to simultaneous exposure to multiple anthropogenic selection pressures are rare and often generate conflicting results. We used an in situ reciprocal transplant design within a quantitative genetic framework to examine how adaptive evolution and phenotypic plasticity contribute to the persistence of an amphibian population inhabiting an environment characterized by high levels of multiple toxic trace elements. We found evidence of phenotypic divergence that is largely consistent with local adaptation to an environment contaminated with multiple chemical stressors, tied to potential trade‐offs in the absence of contaminants. Specifically, the population derived from the contaminated environment had a reduced risk of mortality and greater larval growth and in the contaminated environment, relative to offspring from the naïve population. Further, while survival in the uncontaminated environment was not compromised in offspring from the contaminant‐exposed population, they did show delayed development and reduced growth rates over larval development, relative to the naïve population. We found no evidence of reduced additive genetic variation in the contaminant‐exposed population, suggesting long‐term selection in a novel environment has not reduced the evolutionary potential of that population. We also saw little evidence that past selection in the ASH environment had reduced trait plasticity in the resident population. Maternal effects were prominent in early development, but we did not detect any trends suggesting these effects were associated with the maternal transfer of toxic trace elements. Our results demonstrate the potential for adaptation to multiple contaminants in a wild amphibian population, which may have facilitated long‐term persistence in a heavily impacted environment.

## 1 INTRODUCTION

Human‐induced environmental change has modified ecosystems on a global scale and presents challenges for the persistence of wildlife populations (Vitousek et al., 1997). Specifically, habitat degradation can create novel, stressful environments beyond the range of conditions to which many organisms are adapted (Sih et al., 2011). When faced with novel stressors, populations can decline and go locally extinct if fitness is severely reduced (Butchart et al., 2010). Alternatively, organisms can respond by migrating to more favorable habitats or improving their fitness in the environment via local adaptation or phenotypic plasticity. These responses are not mutually exclusive, and current theories offer conflicting predictions about how interactions among them could influence the evolutionary process (Ghalambor et al., 2007; Levis & Pfennig, 2016). Specifically, adaptive plasticity is predicted to either hinder (Ancel, 2000; Price et al., 2003) or facilitate (Chevin et al., 2010; Lande, 2009; Waddington, 1953; West‐Eberhard, 2003) local adaptation.

One of the most pervasive contributors to human‐induced environmental change is the chemical contamination of habitats (Nelson, 2005). Laboratory studies suggest that chemical stressors can negatively affect traits associated with survival and fitness (Carey & Bryant, 1995; Egea‐Serrano et al., 2012). Evolutionary responses to chemically contaminated environments can facilitate the persistence of populations by reducing the fitness costs typically incurred when local conditions differ from those historically experienced (Meyer & Di Giulio, 2003). Specifically, if selection for tolerance to chemical contaminants leads to local adaptation, we would expect the selected population to have greater fitness under those conditions than a naïve population (Hereford, 2009; Kawecki & Ebert, 2004). However, such adaptation is often associated with life history trade‐offs (Futuyma & Moreno, 1988; Stearns, 1992) that can have implications for long‐term population viability. For example, in the absence of the chemical stressor, populations that have evolved tolerance to those stressors can coincide with reductions in fitness (Shirley & Sibly, 1999; Xie & Klerks, 2004) and fitness‐related traits, including resistance to infection (Hua et al., 2017), growth (Shirley & Sibly, 1999), and development (Xie & Klerks, 2004). Thus, while populations may adapt to chemical contaminants, the costs incurred could negatively affect their long‐term viability.

Genetic adaptation to novel environments is contingent on sufficient genetic variation in the traits under selection. A number of studies have reported extensive within‐ and among‐population variation in susceptibility to environmental contaminants, which suggests there is genetic variation in contaminant tolerance in wild populations (Barata et al., 2002; Bridges & Semlitsch, 2000; Flynn et al., 2015, 2019; Lance et al., 2012, 2013; Metts et al., 2012; Piola & Johnston, 2006). However, few studies have determined whether this variation is heritable, a product of maternal effects, and/or a result of phenotypic plasticity or epigenetic responses to chemical stressors (but see Bridges & Semlitsch, 2011; Klerks & Levinton, 1989; Klerks & Moreau, 2001; Posthuma et al., 1993; Räsänen et al., 2003; Roelofs et al., 2006; Semlitsch et al., 2000). Further, when heritability is measured, it is often in the context of laboratory studies under artificial conditions that do not reflect the complex environments experienced by wild populations (reviewed in Weigensberg & Roff, 1996). Incorporating reciprocal transplant and quantitative genetic approaches in wild populations under field conditions is necessary to provide more realistic estimates of phenotypic and genetic variation in chemical tolerance and life history traits, as well as potential trade‐offs associated with tolerance.

In addition to genetic adaptation, phenotypic plasticity may allow populations to persist in the presence of novel environments. Plasticity can allow individuals to produce a different phenotype in response to environmental conditions (West‐Eberhard, 2003). Plastic responses to environmental variables such as predators (Freeman & Byers, 2006), changing temperatures (Walsh et al., 2014) and contaminants (Hua et al., 2013) are widespread. In some cases, exposure to stressors, such as pesticides or metals, early in development, induces a plastic phenotype that has increased tolerance to that stressor later in life (Herkovits & Pérez‐Coll, 2007; Hua et al., 2013; Lauren & McDonald, 1987; Tate‐Boldt & Kolok, 2008). If the new phenotype improves fitness in the novel environment, then plasticity can provide an initial rescue before genetic adaptation and evolutionary rescue (Bell & Collins, 2008; Chevin et al., 2010). Plasticity may also facilitate local adaptation if a broader array of phenotypes are produced for selection to act on, especially if maladaptive phenotypes result and are selected against (Ghalambor et al., 2007, 2015). However, there remains uncertainty over the role of phenotypic plasticity in facilitating genetic adaptation (Ghalambor et al., 2007) and there may be costs and life history trade‐offs associated with plasticity (Agrawal et al., 2002; Gervasi & Foufopoulos, 2008; Relyea, 2002).

Amphibians are an ideal system to assess the relative importance of adaptation and plasticity in response to anthropogenic stressors. Substantial theoretical (Werner, 1986; Wilbur & Collins, 1973) and empirical (Berven, 1990; Smith‐Gill & Berven, 1979) work has examined how larval amphibians alter their growth and/or development rates in response to local conditions. Amphibians are also susceptible to a number of factors associated with human‐induced environmental change, in part due to their unique physiological constraints, limited dispersal capability, and high site fidelity (Blaustein et al., 1994). Because many amphibians rely on aquatic habitats for larval development and reproduction, habitat degradation resulting from chemical contamination is of particular concern. Trace element (TE) contaminants, including metals and metalloids, are especially common in aquatic environments and can lead to multi‐generational exposure because, as elements, they do not degrade (Linder & Grillitsch, 2000). Exposure to elevated TEs in aquatic environments is associated with a broad range of detrimental effects (Flynn et al., 2015; Hopkins et al., 1999; Lance et al., 2013; Metts et al., 2012; Peles, 2013; Rowe et al., 2001; Snodgrass et al., 2004), but despite these immediate negative impacts on fitness, some populations continue to thrive in the presence of these persistent stressors (Weis & Weis, 1989).

Here, we focused on an environment impacted by coal fly ash (hereafter “coal ash”), a globally pervasive source of TEs. Coal ash is often stored in surface impoundments (USEPA, 2010), which also serve as permanent sources of freshwater that are attractive to wildlife. Wildlife exposed to the ash in these impoundments can experience reductions in reproduction, survival, and development (Metts et al., 2012; Raimondo et al., 1998; Rowe et al., 2001). Given the susceptibility of amphibian larvae to environmentally relevant levels of TEs and the substantial variation in TE tolerance within and among populations (Flynn et al., 2019), environmental exposure to coal ash could effectively select for TE tolerance. We used a quantitative genetic breeding design in an embryonic common garden study followed by a larval reciprocal transplant study. We characterized patterns of phenotypic plasticity between a population inhabiting a coal ash disposal site with a nearby population inhabiting an environment without a history of contamination to (i) assess evidence of local adaptation and life history trade‐offs associated with a coal ash‐contaminated environment and (ii) estimate quantitative genetic variation in life history traits and their plasticity to make inferences about past selection and to assess the future evolutionary potential of the population. Given the widespread within‐population variation in amphibian tolerance to TEs (Flynn et al., 2015, 2019; Lance et al., 2012, 2013) and the fact TEs can negatively impact survival and fitness‐related traits, we predicted that a population subjected to toxic levels of TEs for decades would exhibit elevated fitness in that environment relative to a nearby population without a history of exposure. We also expected that key life history traits in the population resident in this contaminated environment would not be as negatively impacted by the chemical stressors as the TE naïve population. Lastly, we predicted that this adaptive divergence would be associated with trade‐offs in the absence of toxic TEs and with reduced genetic variation in life history traits due to stabilizing or directional selection for tolerance.

## 2 METHODS

### 2.1 Study species

Southern toads (*Anaxyrus terrestris*, Bonnaterre) are widely distributed across the southeastern United States and can be one of the most abundant anuran species in wetlands within their range (Bennett et al., 1980). *Anaxyrus terrestris* are indiscriminate breeders, using a variety of ephemeral, permanent, natural, and constructed aquatic habitats (Gibbons & Semlitsch, 1991; Hopkins et al., 1997; Wright & Wright, 1949), and they remain common in areas even in the face of severe habitat destruction and urbanization (Bartlett & Bartlett, 1999). They can live up to ten years (Ashton & Ashton, 1988) and reach sexual maturity in 2–3 years (D. Scott, personal communication).

### 2.2 Study populations and sites

The study sites consisted of a coal ash disposal site (ASH) and a large (>10 acre) temporary wetland, considered the reference site (REF), located ~3.0 km from ASH. Both sites are located on the United States’ Department of Energy Savannah River Site in Aiken County, South Carolina. The ponds at the ASH site were created in the 1950s to manage waste from a nearby coal‐fired power plant (USEPA, 2007). While *A*. *terrestris* use these basins for reproduction, a relatively low number of metamorphosed individuals are generally observed at the site (Rowe et al., 2001). The water chemistry at the ASH site differs from natural wetlands (including REF), having exceedingly high specific conductance, more basic pH (Table S1), and low levels of dissolved organic carbon (Rowe et al., 2001). The water, sediment, and biofilms at the ASH site also contain highly elevated levels of numerous TEs, including arsenic, nickel, and selenium (see Table S2; Roe et al., 2005).

Adult *A*. *terrestris* living in and around the ASH site (i.e. the ASH population) have elevated levels of many TEs, relative to reference populations (Table S3; Text S1; Hopkins et al., 1999; Metts et al., 2012), which can be transferred maternally to offspring (Flynn et al., 2019; Hopkins et al., 2006). Adults did not differ in size between populations (Figure S3; Text S1). We assume that there is little to no mixing between animals from REF and ASH (i.e. independent populations) based on estimates that ~99% of individuals reside within 1 km of their breeding site (Semlitsch, 2008).

### 2.3 Artificial fertilization design and methods

We captured adult toads at drift fences at both sites as they were migrating to breed between 3/31/14 and 4/10/14. We recorded mass and snout‐vent length (SVL) for all adults, which were used to calculate body condition indices for each animal. Adults from each population were bred using artificial fertilization methods to produce full‐sibling/half‐sibling families (see Text S2 for detailed methods). We used a buffered 4% MS‐222 solution to euthanize males and females, prior to removing testes and immediately after oviposition, respectively. The overall breeding design consisted of 32 sires and eight dams per site, divided into four breeding blocks consisting of 8 sires and two dams each (Figure S1). Breeding within each block was fully crossed, which resulted in 16 full‐sibling families per block, 64 full‐sibling families per population, and 128 families total. Of these families, four crosses from the REF population did not produce any viable offspring (see Table S4). This breeding design maximized the power to estimate sire effects but limited statistical power to estimate dam effects. We preserved a subset of ten eggs from each female (except for one female from the REF population due to low reproductive output) to take scaled photographs to determine mean egg size for each clutch using the program ImageJ (Schneider et al., 2012).

### 2.4 Embryonic common garden study

We reared embryos in the University of Georgia's Savannah River Ecology Laboratory's Animal Care Facility before deploying larvae to the field. We removed any obviously dead or nondeveloping embryos 24‐h postfertilization and then assigned the remaining embryos to two treatments two days postfertilization (ASH: 4/17/15 and REF: 4/20/15). Treatments consisted of water collected from either the REF or the ASH pond. We used a full factorial design that included two source populations × 64 families (generated from eight dams and 32 sires from each population) × two water treatments × three replicates, for a potential of 768 experimental units. However, some crosses did not produce enough viable offspring to fill any or all of the replicates for a given treatment (see Table S4), which resulted in a total of 727 experimental units. Each replicate corresponded to a shelving unit in our climate‐controlled Animal Care Facility. Previous research in this room has shown that the temperature is consistent vertically but can vary slightly based on distance from the door/HVAC unit. Each family‐treatment combination was represented once in each block. There were too few successfully fertilized eggs from some clutch × treatment combinations to fill any or all of the replicates (see Table S4A). We reared groups of embryos (range: 8–60) in 0.5‐L plastic containers containing 400 ml of water sourced from either the REF or ASH sites. By five days postfertilization, all embryos had reached Gosner stage 25, (GS25; Gosner, 1960), at which time we assessed survivorship before pooling all replicates within a family × treatment and haphazardly sampling ten larvae (in some cases fewer when survivorship was low for a family × treatment group) to determine mean size (i.e. total length) at GS25 for each family × treatment combination in ImageJ from scaled photographs. Water samples were taken from a subset of experimental units at the start and end of the embryonic study to quantify major cations and TEs in the test solutions (Table S5).

### 2.5 Larval reciprocal transplant study

Subsets of six larvae (fewer in some cases due to differential fertilization success and mortality; see Table S4B) from each family × rearing solution combination from the embryo trial were carried through to the field portion of the study. We individually reared these larvae to metamorphosis in situ in field enclosures at the site (ASH or REF) corresponding to the rearing solution they experienced in embryonic development (e.g., individuals reared through embryonic development in water collected from the REF environment were continued on in the REF environment in the field). We designed the enclosures to allow individual rearing of larvae and regular exchange of water (see Figure S2 for details). The general design consisted of a 26.5‐L plastic bin (58 × 41 × 15 cm) into which six smaller 1‐L plastic containers (14.5 × 14.5 × 11.5 cm) were nested. We removed the centers of lids and container bottoms of both the small and large containers and replaced them with nonmetal screen to keep larvae in and exclude predators, while allowing for exchange of water, suspended sediments, and air with the surrounding environment. The enclosures were deployed two weeks prior to the start of the field trial to allow the interior of the containers to be colonized with resident algae and biofilms to provide a food source for developing larvae.

Field enclosures were grouped into six spatial blocks in each environment, where one larva from each family (i.e. one replicate) was represented in each block. We used a random number generator to assign families to containers within each block. Due to low fertilization and survivorship to the larval stage in several families from the REF population and an experimental error, offspring from two breeding blocks were not used in the field study. This resulted in an unbalanced design for the field portion of the study, with 64 families represented from the ASH population and 32 from REF. Combined with the variable numbers of offspring produced among crosses, the total number of larvae used in each population × site combination was 184 and 166 for the REF population in REF and ASH sites (350 total), respectively, and 383 and 345 for the ASH population in REF and ASH sites (728 total), respectively.

We transferred larvae to field enclosures two days after all surviving larvae reached GS 25 (ASH: 4/25/14, REF: 4/28/14). We measured all individuals for early larval size (total length), early larval growth, time to metamorphosis (days), size at metamorphosis (SVL and mass), mean growth rate (mg/day), and mortality. Initially, we made observations three times per week at which time we exchanged water by gently lifting each enclosure up until only ~1 cm of water remained and setting it back down. After the first larvae began developing visible rear legs, we began checking enclosures daily. We also took scaled photos of individual larvae after seven days in the field (ASH: 5/2/14, REF: 5/5/14) and again at 21 days (i.e. 14 days later; ASH: 5/16/14, REF: 5/19/14) to determine early size and growth using ImageJ. Individuals were determined to have metamorphosed upon the emergence of at least one forelimb (GS42). All metamorphic individuals were brought back to the laboratory and maintained at 23℃ (±1.5℃) in 0.5‐L plastic containers with an unbleached paper towel dampened with water from the individual's field container. When mortality was observed, the individuals were removed and not replaced.

We terminated the field study on 08/07/14 (101st and 104th day in the field for REF and ASH populations, respectively) when only eight larvae remained unmetamorphosed and had shown no signs of further development for several weeks.

Water temperature was recorded hourly using sealed temperature loggers placed in the bottom of every other field enclosure to obtain a temperature profile for every spatial block within each environment (Figure S5). On 5/2/14 and 6/9/14, we measured pH and specific conductance (μS/cm) in two randomly selected bins and the surrounding water for a subset of spatial blocks (YSI Pro Plus Quatro Field Cable). At the same time, we collected two 14 ml water samples at ~4 cm below the water surface (one inside and one outside the bins), in every other spatial block, for subsequent analyses of total levels of major elements and TEs (Table S6).

### 2.6 Metamorphic toad processing protocol

After metamorphic individuals were returned to the laboratory, we observed them daily to monitor tail resorption. Two days after individuals reached GS46 (GS46 was determined as <1.0 mm of tail remaining), animals were gently blotted dry before being weighed (±0.01 mg) and measured for SVL to the nearest 0.5 mm using a ruler. We euthanized individuals within 2 min of initial handling by immersion in a 4% solution of MS‐222.

### 2.7 Sample prep and elemental analysis

To prepare water samples collected in the laboratory and field for analysis, we added 140 μl of trace metal‐grade nitric acid (HNO_3_) to the 14 ml of each sample in trace element‐free certified 15 ml conical tubes (VWR^®^) to yield a final concentration of 1% HNO_3_. Acidified samples were run on an inductively coupled plasma mass spectrometer (ICP‐MS, Nexion 300X ICP‐MS; Perkin Elmer) for TE (aluminum [Al], arsenic [As], barium [Ba], beryllium [Be], cadmium [Cd], cobalt [Co], copper [Cu], nickel [Ni], tin [Sb], selenium [Se], strontium [Sr], thorium [Th], uranium [U], vanadium [V], and zinc [Zn]) and major cations (sodium [Na], calcium [Ca], magnesium [Mg], and potassium [K]).

We prepared liver samples collected from parental toads by freeze drying and weighing to the nearest 0.01 mg before digesting in tubes with 300 μl HNO_3_ placed on a heat block at 80℃ for 2 h. We also digested reference standards (TORT‐3, National Research Council Canada) and blanks for quality control and determination of minimum detection limits. Digested samples were diluted with ultrapure water before analyzing on ICP‐MS (3.33% HNO_3_ final sample concentration). Due to small dry masses associated with the tissue samples, only a subset of elements was analyzed.

### 2.8 Statistical and quantitative genetic analyses

All statistical analyses were carried out in R (R Core Development Team, 2013). We examined differences in water quality (pH, conductivity, Na, Ca, Mg, and K) and measured TE concentrations between environments and among spatial blocks within environments using MANOVAs followed by univariate ANOVAs. We also performed principal component analysis (PCA; *prcomp* in *stats* package; R Core Development Team, 2013) with scaling, centering, and varimax rotation to provide a more comprehensive analysis of differences in water chemistry within and between rearing environments (Figure S4; Table S7).

### 2.9 Characterizing effects of population and environment on phenotype

We assessed differences in embryonic survival and larval and metamorphic traits between populations and rearing environments by fitting generalized linear mixed models. Each model included fixed effects for population of origin (hereafter “population”), rearing environment (hereafter “environment”), and their interaction.

Embryonic survival was modeled as a bivariate response of successes and failures using a bivariate distribution (family = “multinomial2”) in the R package MCMCglmm using weak, noninformative parameter expanded priors (Hadfield, 2010, 2019; Text S3). The random effects structure included terms for sire, dam, sire x dam, and experimental unit. We also included the initial number of viable embryos in each experimental unit at the start of the study as a covariate, given that it differed among families and experimental units due to variation in fertilization success and can influence early survival (Lance et al., 2013). Initially, mean embryo size for each clutch was also included as a covariate but removed from final models as it was not significant (pMCMC = 0.310) and did not improve model fit.

When testing for phenotypic differences in larval and metamorphic traits, the random effect structure included within‐environment spatial block and environment‐specific terms for sire, dam, and sire × dam (*glmer* in *lme4* package; Bates et al., 2015; Text S3). We did not include water temperature in our models as we determined spatial block was a significant predictor of temperature and therefore opted to use block alone as it accounted for spatial differences in temperature within environment as well as other microenvironmental differences. Continuous response variables were visually inspected to confirm data followed an approximately Gaussian distribution. Larval mortality was modeled as a binary response (1 = died prior to metamorphosis, 0 = survived to metamorphosis or end of study) using a binomial (logit‐link) distribution. Significance of fixed effects was determined using Wald‐*χ* ^2^ tests with Type III error (*car* package: ANOVA; Fox & Weisberg, 2011), and pairwise comparisons of means were performed using estimated marginal means (*emmeans* package).

We also used Cox's proportional hazard models to test how probability of larval mortality varied with population and rearing environment. These semi‐parametric analyses incorporate a binary value for the event (i.e. 0 = survived, 1 = died) and the time to the event (i.e. death). First, we examined the overall effects of population, environment, and their interaction on mortality risk with a model including fixed effects for rearing environment, population of origin, and their interaction and spatial block within environment as a clustering term. Individuals that survived to the end of the study but had not metamorphosed (*n* = 8) were included as censored observations. The initial model indicated that hazards deviated substantially from the assumption of proportionality (i.e. varied between environments and over time) based on inspection of survival curves, Schoenfeld residual plots, and the corresponding goodness‐of‐fit tests (Schoenfeld, 1982; Therneau, 2021; Therneau et al., 2021). To mitigate this issue, we split the dataset into two time intervals (days 0–30 and days 31–end of study) based on the above observations and the need to retain sufficient data for model convergence in both intervals. This resulted in two initial models (one for each time interval) that better met the proportional hazards assumption (i.e. based on Schoenfeld residual plots and associated tests). We also examined how the splitting the data at other time points affected the results. Briefly, models using splits at 21, 35, and 45 days produced qualitatively similar results as the day 30 split, with hazard estimates that differ by less than 10%. To test how the probability of mortality differed between populations within a given environment, we subdivided the data by environment and time interval. Each of these subsequent models included population as a fixed effect and spatial block as a clustering term. This approach generates hazard ratios associated with probability of mortality for a given population in a given environment that have a convenient direct interpretation. Significant hazard ratios <1 show that the probability of mortality is reduced for the ASH population relative to the REF population or environment, while those >1 show probability mortality was elevated for the ASH population, relative to the REF population. For example, a hazard ratio of 0.5 associated with the population term in the model would suggest that the probability of mortality of the ASH population in that environment was half that of the REF population.

### 2.10 Estimating quantitative genetic parameters

To assess quantitative genetic parameters associated with population‐specific responses of traits in each of the environments, we conducted additional analyses modeling embryonic, larval, and metamorphic traits. Specifically, for each population, we fit separate generalized linear mixed models in a Bayesian framework using Markov Chain Monte Carlo sampling in the R package *MCMCglmm* (Hadfield, 2010). For continuous traits, we used weakly informative priors with inverse‐Gamma distributions (see Text S3) and optimized each model to ensure adequate mixing and negligible autocorrelation (Hadfield, 2019). Larval mortality models were fit using probit distribution (family = “threshold”), while embryonic survival was modeled as a bivariate trait of dead and surviving embryos (family = “multinomial2”). We included initial number of viable embryos as a covariate in embryonic survival models as it improved precision of variance estimates and improved model mixing. These models included only the intercept as a fixed effect, with a random effects structure including sire, dam, sire × dam, and spatial block (Text S3). We estimated causal variance components as follows:

$$V_{P}=\sigma_{\mathrm{sire}}^{2}+\sigma_{\mathrm{dam}}^{2}+\sigma_{\mathrm{sire}:\mathrm{dam}}^{2}+\sigma_{\mathrm{block}}^{2}+\sigma_{\mathrm{residual}}^{2}$$

$$\sigma_{\mathrm{sire}}^{2}=\frac{1}{4}V_{A}$$

$$\sigma_{\mathrm{dam}}^{2}=\frac{1}{4}V_{A}+V_{M}$$

$$\sigma_{\mathrm{sire}:\mathrm{dam}}^{2}=\frac{1}{4}V_{D}$$

$$\sigma_{\mathrm{residual}}^{2}=\frac{1}{2}V_{A}+\frac{3}{4}V_{D}+V_{E}$$

Additive genetic (*V*_A_), maternal (*V*_M_), and nonadditive genetic (*V*_D_) contributions to total phenotypic variance of traits were population‐ and rearing environment‐specific, with total phenotypic variation ($V_{P}$) expressed as the sum of variances for all random effects in a given model, including residual variance. We calculated the proportion of phenotypic variance explained by each quantitative genetic component by dividing *V*_A_, *V*_M_, and *V*_D_ by *V*_P_, yielding estimates of narrow‐sense heritability (*h* ^2^), nongenetic maternal, and nonadditive genetic contributions, respectively. These parameters were also estimated for the plasticity of traits using the random terms for the interactions of rearing environment with sire, dam, and sire × dam. The heritability of plasticity, $h_{\mathrm{pl}}^{2}$, was calculated following the approach of Becker (1964) and Scheiner and Lyman (1989):

$$h_{\mathrm{pl}}^{2}=\frac{{4\sigma }_{\mathrm{SXE}}^{2}}{\sigma_{P}^{2}}$$

where $\sigma_{\mathrm{SXE}}^{2}$ is the variance associated with sire (*S*) between environments (*E*). Estimates of maternal and nonadditive genetic contributions to between environment plasticity were similarly determined by substituting (dam × environment – sire × environment) and 4 (sire × dam × environment), respectively into the numerator. All credible intervals for Bayesian analyses are given as 95% highest posterior density intervals (HPDI). We used the MCMC‐derived slopes and *p*‐values from model “environment” terms (i.e. β‐estimates) to determine statistical significance, direction, and slope of the plastic response of traits to rearing environment.

## 3 RESULTS

### 3.1 Water quality and chemistry

The aquatic environments at the two sites differed markedly in their water chemistries (MANOVA: *F*_1,24_ = 7266.2, *p* < 0.001), but there was little variation among spatial blocks within environment (MANOVA: *F*_4,24_ = 1.2, *p* = 0.268). Water pH, specific conductance, Ca, Na, K, and Mg were always greater in ASH than REF, and there was relatively little within‐environment variation (Figure S4; Table S1 for full account). Similarly, TE levels in ASH were either greater than or not different from REF, with the exception of Zn (Table S2). Principal components analysis revealed the two environments differed most along PC1, which was associated with pH, conductivity, As, Ba, Ni, Se, and Sr (see Figure S4; Table S7). Temperature profiles differed among environments with ASH being consistently warmer and having less daily fluctuation in temperature than REF (Figure S5).

### 3.2 Phenotypic plasticity and population divergence

We quantified phenotypic divergence between populations in the following key amphibian life history traits over the course of embryonic and larval development: embryo size, embryo survival, early larval growth, time to and size at metamorphosis, growth rate over larval development, and probability of larval mortality. The effects of rearing environment, population of origin, and their interaction varied by trait, but overall suggest local adaptation of populations to their natal aquatic environments as detailed below.

Embryo size differed significantly between populations (*F*_1,149_ = 184.41, *p* < 0.001), such that embryos from ASH dams were larger than those from REF (Figure S6), even after accounting for variation in dam size (dam size: *F*_1,149_ = 3.78, *p* = 0.054). Survival through embryonic development was not influenced by water source (*p* = 0.857), population of origin (*p *= 0.426), or their interaction (*p* = 0.503; Figure S7). Initial number of embryos in experimental units had a significant positive effect on survival (*p* = 0.024), but mean embryo size did not. Size at GS25 was affected by rearing solution (Wald‐*χ* ^2^ = 24.51, *p* < 0.001), but not by population of origin (Wald‐*χ* ^2^ = 0.96, *p* = 0.327, Figure S7). However, there was a significant population by rearing solution interaction (Wald‐*χ* ^2^ = 23.95, *p* < 0.001) such that larvae from the ASH population were of similar size in both solutions, but REF larvae were larger in the ASH solution. This suggests the effects of rearing solution on size at GS25 are dependent on population of origin.

**FIGURE 1:**
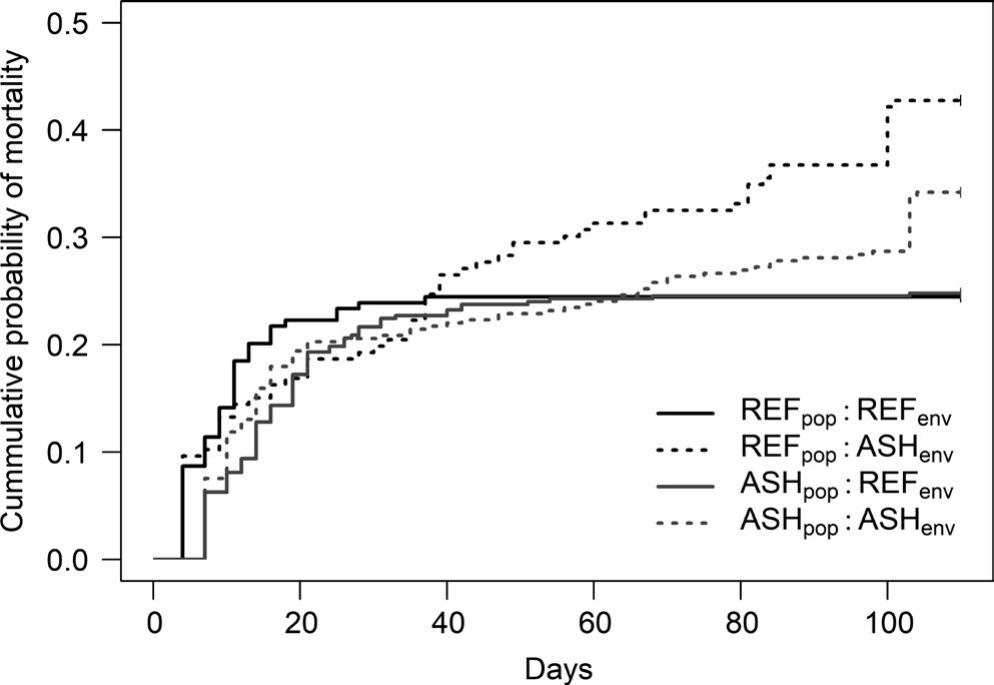
Survival curves describing the cumulative probability of mortality over the course of the study. In the REF environment (solid lines), both populations had a similar probability of mortality. The ASH environment (dashed lines) increased the probability of mortality; however, the extent of this increase was dependent on the population of origin and interval of time over which observations were made

Survival through larval development in the REF environment was high and nearly identical for both populations (75.5% for REF vs. 75.1% for ASH). In the ASH environment, survival to metamorphosis was significantly reduced for both populations (57% for REF vs. 66% for ASH), although the difference in survival between populations was not statistically significant when only considering mortality. Using Cox proportional hazard models incorporating not only mortality, but when mortality occurred, we found that risk of mortality was not affected by population or rearing environment during the first 30 days of development, but over the remaining 74 days, a significant population × environment interaction suggested the mortality risk of the ASH population in the ASH environment was lower than expected based on main effects of population and environment (hazard ratio = 0.13, *p* = 0.033; Figure 1). Therefore, we split the dataset by rearing environment for each time interval for further analysis. These models showed the risk of mortality did not differ between populations in either environment during the first time interval (i.e. days 0–30). When examining the second time interval (i.e. days 31–104), the risk of mortality only differed in the ASH environment, where the ASH population was 30% less likely to experience mortality than the REF population (hazard ratio = 0.31, *p* < 0.001).

**FIGURE 2:**
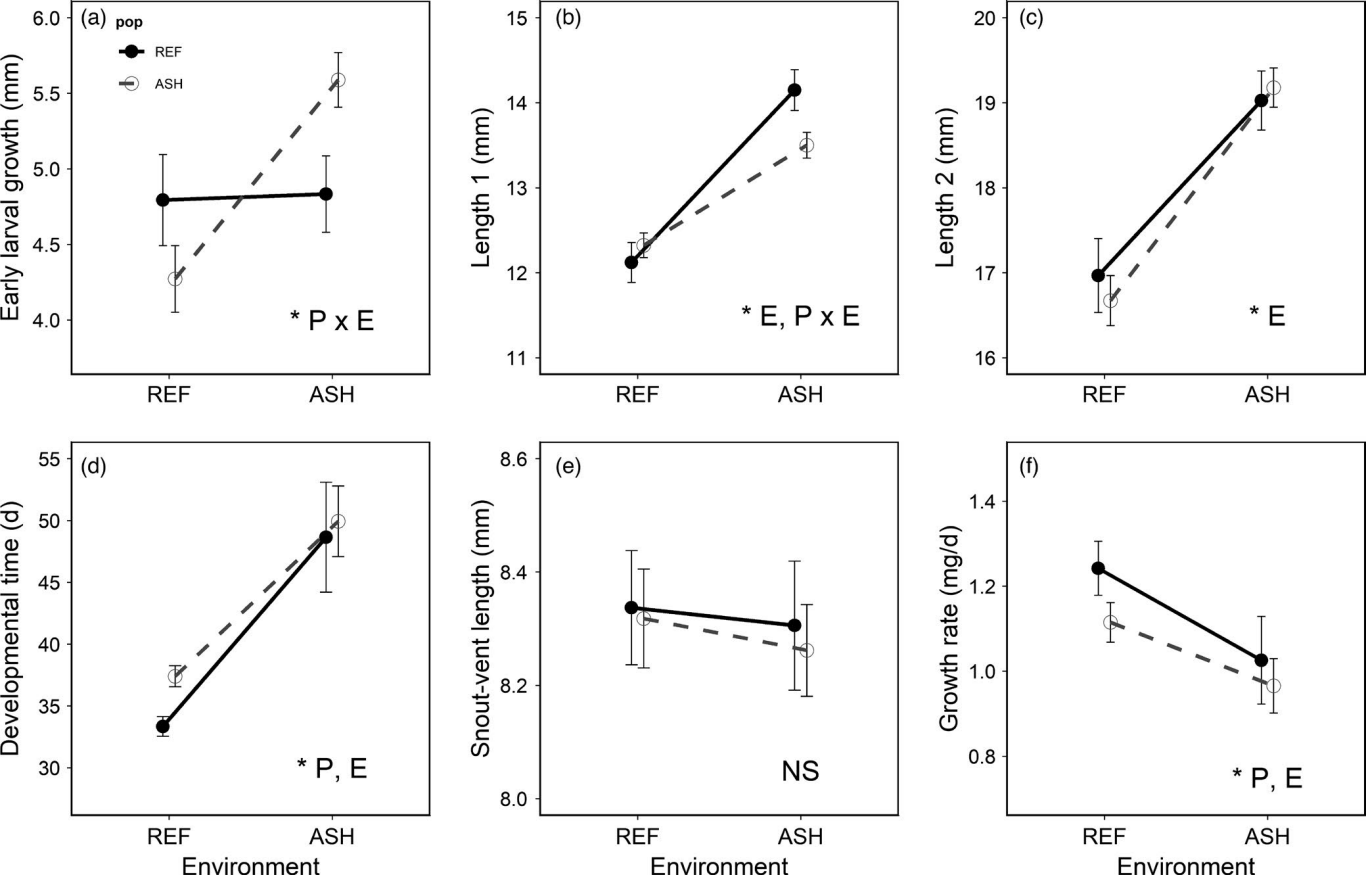
Plots of larval (a–c) and metamorphic (d–f) trait means for REF (black) and ASH (gray) populations in each environment. Panel a shows growth over 14 days of early larval development (i.e. day 21–day 7). Panels b and c are total larval length at 7 and 21 days of development, respectively. Panels d–f show time required to reach metamorphosis (ttm), snout‐vent length at metamorphosis (SVL), and growth rate over entire larval period (i.e. mass at metamorphosis/time to metamorphosis), respectively. Asterisks and accompanying letters in bottom right of panels denote significant population (P), environment (E), and population × environment effects. Error bars denote 95%‐CI

The effect of rearing environment on early larval growth (i.e. change in size between days 7 and 21) was dependent on population of origin (Wald‐*χ* ^2^ = 31.85, *p* < 0.001). While REF larvae grew similarly in both environments, individuals from the ASH population displayed considerable plasticity (Figure 2a). Specifically, ASH larvae grew more rapidly in the ASH environment and less rapidly in the REF environment, relative to individuals from the REF population. Initially, larvae from both populations were larger in the ASH environment than REF (day 7: Wald‐*χ* ^2^ = 40.54, *p* < 0.001; day 21: Wald‐*χ* ^2^ = 8.71, *p* = 0.003, Figure 2b). However, ASH larvae reared in the ASH environment were smaller than REF larvae reared in ASH at day 7 (*p* = 0.036; Figure 2b) but had caught up in size by the end of this period (day 21: *p* = 0.531; Figure 2c).

Being reared in the ASH environment delayed development for both populations (Wald‐*χ* ^2^ = 8.89, *p* = 0.003). However, in the REF environment, the REF population developed more rapidly than did the ASH population (*p* = 0.030; Figure 2d), with the slopes of reaction norms trending toward being significantly different between populations (Wald‐*χ* ^2^ = 3.33, *p* = 0.068). Size at metamorphosis (SVL) did not differ by population of origin (Wald‐*χ* ^2^ = 0.12, *p* = 0.727) or rearing environment (Wald‐*χ* ^2^ = 0.09, *p* = 0.768), and there was no evidence of a population by environment interaction (Wald‐*χ* ^2^ = 0.004, *p* = 0.947, Figure 2e). Though not reported, we ran models using mass at metamorphosis as the response variable, which generated similar results.

Growth rate over the entire developmental period (mg/day) was significantly greater in the REF population (Wald‐*χ* ^2^ = 6.37, *p* = 0.011) and the REF environment (Wald‐*χ* ^2^ = 5.40, *p* = 0.020, Figure 2f). Keeping in line with observed differences in time to metamorphosis, pairwise comparisons showed that the growth rate of the REF population was significantly greater than the ASH population only when reared in the REF environment (Figure 2f).

### 3.3 Quantitative genetic variation in life history traits and their plasticity

Additive genetic, maternal, and nonadditive genetic effects are reported in Table 1 (see Table S8 for raw variances). Most traits showed low to moderate heritability; however, heritability estimates for survival through embryonic development and probability of metamorphosis were negligible (i.e. low estimates with credible intervals including zero; Table 1; see Table S8 raw variances). Across traits, estimates of nonadditive genetic variance were generally similar to those for heritability (Table 1). By contrast, maternal effects not overlapping zero were only detectable in traits tied to embryonic development, namely embryonic survival and size at GS25 (Table 1).

**TABLE 1:** Proportion of phenotypic variance explained by causal variance components for embryonic and larval traits. Estimates obtained from population and environment‐specific posterior‐mean estimates (95% HPDI). Initial number of embryos present in each experimental unit was included as a covariate in models of embryonic survival. For early larval growth, the response variable was the difference in total length of larvae between days 7 and 21 of the field study. Growth rate was analyzed using mass at metamorphosis as the response variable with time to metamorphosis as a covariate

Pop.	Env.	Embryo survival			Length (GS25)		
		*h* ^2^	mat	dom	*h* ^2^	mat	dom
REF	REF	0.11 (0.00–0.49)	0.38 (0.06–0.76)	0.06 (0.00–0.32)	0.56 (0.23–1.0)	0.23 (0.00–0.61)	0.49 (0.21–0.85)
	ASH	0.06 (0.00–0.81)	0.45 (0.15–0.81)	0.05 (0.00–0.25)	0.48 (0.18–0.93)	0.32 (0.01–0.70)	0.38 (0.15–0.72)
ASH	REF	0.05 (0.00–0.25)	0.51 (0.21–0.82)	0.05 (0.00–0.25)	0.44 (0.16–0.86)	0.35 (0.05–0.71)	0.33 (0.13–0.59)
	ASH	0.08 (0.00–0.39)	0.42 (0.14–0.76)	0.04 (0.00–0.20)	0.42 (0.17–0.76)	0.28 (0.01–0.63)	0.36 (0.15–0.62)

Pop.	Env.	Early growth (mm/day)			ttm		
		*h* ^2^	mat	dom	*h* ^2^	mat	dom
REF	REF	0.13 (0.02–0.43)	0.06 (0.00–0.40)	0.27 (0.04–0.78)	0.29 (0.07–0.74)	0.12 (0.00–0.64)	0.28 (0.07–0.65)
	ASH	0.23 (0.04–0.72)	0.06 (0.00–0.47)	0.48 (0.06–1.26)	0.27 (0.03–0.89)	0.06 (0.00–0.51)	0.24 (0.02–0.82)
ASH	REF	0.10 (0.01–0.31)	0.08 (0.00–0.29)	0.17 (0.00–0.50)	0.31 (0.10–0.67)	0.03 (0.00–0.23)	0.30 (0.10–0.63)
	ASH	0.13 (0.04–0.32)	0.04 (0.00–0.20)	0.16 (0.04–0.37)	0.11 (0.02–0.32)	0.02 (0.00–0.14)	0.17 (0.03–0.48)

Pop.	Env.	svl			Growth rate (mg/day)		
		*h* ^2^	mat	dom	*h* ^2^	mat	dom
REF	REF	0.44 (0.08–1.0)	0.24 (0.00–0.80)	0.34 (0.07–0.76)	0.48 (0.07–1.2)	0.35 (0.00–0.88)	0.32 (0.05–0.74)
	ASH	0.44 (0.08–1.1)	0.24 (0.00–0.81)	0.37 (0.07–0.83)	0.49 (0.08–1.2)	0.29 (0.00–0.85)	0.38 (0.07–0.88)
ASH	REF	0.35 (0.12–0.68)	0.10 (0.00–0.39)	0.34 (0.13–0.63)	0.41 (0.13–0.83)	0.21 (0.00–0.58)	0.30 (0.10–0.59)
	ASH	0.40 (0.15–0.77)	0.09 (0.00–0.37)	0.51 (0.20–0.91)	0.40 (0.13–0.79)	0.17 (0.00–0.50)	0.34 (0.11–0.64)

Pop.	Env.	Probability of mortality					
		*h* ^2^	mat	dom			
REF	REF	0.20 (0.00–0.96)	0.04 (0.00–0.60)	0.20 (0.00–0.85)			
	ASH	0.17 (0.00–0.69)	0.04 (0.00–0.52)	0.23 (0.00–0.92)			
ASH	REF	0.14 (0.00–0.37)	0.03 (0.00–0.21)	0.30 (0.00–0.87)			
	ASH	0.17 (0.00–0.67)	0.03 (0.00–0.27)	0.14 (0.00–0.58)			

Note

*h*^2^ = additive genetic variance = *V*_A_/*V*_P_, mat = maternal variance = *V*_M_/*V*_P_, dom = nonadditive genetic variance = *V*_D_/*V*_P_, ttm = developmental time in day to metamorphosis, svl = snout‐vent length of metamorphic toads.

There was no evidence additive genetic, maternal, or nonadditive genetic variance differed between populations and rearing environments, although the credible intervals associated with these estimates were large (Table 1). For most traits, heritability estimates were similar among populations and rearing environments. However, for early larval growth, the heritability estimates were nearly twice as high for the REF population reared in the ASH environment compared to other estimates (Table 1). Meanwhile, for time to metamorphosis, the heritability estimate for the ASH population in the ASH environment was less than half that of other estimates (Table 1).

We also estimated the contributions of additive genetic variation to phenotypic plasticity to examine the potential for the evolution of reaction norms and assess whether there was evidence of erosion of genetic variation in plasticity. Additive genetic variation and heritability for plasticity ($h_{\mathrm{pl}}^{2}$) in our analysis are reflected by variation in the slopes of reaction norms among genotypes (i.e. cross‐environmental means by sire; Figure S8). Estimates of $h_{\mathrm{pl}}^{2}$ were low but significant for all traits except embryonic survival and metamorphic probability (Table 2). Estimates of $h_{\mathrm{pl}}^{2}$ were generally similar between populations, although for time to metamorphosis the estimate for the ASH population was ~50% of that for the REF population (Table 2).

**TABLE 2:** Reaction norm slopes (β) and accompanying narrow‐sense heritability ($h_{\mathrm{pl}}^{2}$) MCMC‐estimates obtained from population‐specific plasticity models (see *Methods: statistical and quantitative genetic analyses*). β‐estimates were obtained from population‐specific environment terms and given as trait values in the ASH environment relative to REF. Errors are reported as 95% HPDI, bolded β‐estimates are those for which slope of reaction norms was significantly different from zero

Trait	Pop.	*N*	β‐estimate	$h_{\mathrm{pl}}^{2}$
Embryo survival	REF	303	0.00 (−0.33 to 0.34)	0.03 (0.00–0.14)
	ASH	381	−0.11 (−0.39 to 0.20)	0.02 (0.00–0.10)
Length (GS25)	REF	894	0.01 (−0.02 to 0.03)	0.25 (0.10–0.45)
	ASH	1280	0.00 (−0.02 to 0.02)	0.23 (0.10–0.40)
Length (7d)	REF	313	**0.07 (0.01–0.13)**	0.21 (0.05–0.47)
	ASH	681	**0.04 (0.01–0.08)**	0.21 (0.09–0.38)
Length (21d)	REF	273	0.05 (−0.02 to 0.11)	0.20 (0.05–0.45)
	ASH	557	**0.06 (0.02–0.11)**	0.19 (0.09–0.34)
Early growth (mm)	REF	273	0.05 (−0.02 to 0.12)	0.20 (0.05–0.46)
	ASH	557	**0.06 (0.02–0.11)**	0.19 (0.09–0.35)
ttm	REF	234	**0.15 (0.01–0.30)**	0.19 (0.04–0.51)
	ASH	515	**0.11 (0.00–0.21)**	0.10 (0.03–0.21)
svl	REF	228	0.00 (−0.05 to 0.06)	0.23 (0.04–0.52)
	ASH	497	0.00 (−0.03 to 0.03)	0.23 (0.11–0.41)
Growth rate (mg/day)	REF	384	−0.06 (−0.21 to 0.05)	0.14 (0.02–0.42)
	ASH	768	−0.04 (−0.17 to 0.07)	0.09 (0.02–0.26)
Probability of mortality	REF	384	0.78 (−0.15 to 1.7)	0.04 (0.00–0.15)
	ASH	768	0.41 (−0.30 to 1.2)	0.02 (0.00–0.09)

Note

ttm = developmental time in days to metamorphosis, svl = snout‐vent length of metamorphic toads.

## 4 DISCUSSION

Southern toad populations, differing in their history of exposure to coal ash, diverged in key life history traits when reared in the presence or absence of contaminants. Overall, our results demonstrate phenotypic divergence that is consistent with adaptation to a coal ash‐contaminated environment. Offspring from the ASH population were 30% less likely to die and experienced more rapid larval growth in the contaminated environment relative to the REF population. These observations align with those across a number of taxa, where populations residing in environments impacted by contaminants show greater survival in presence of related chemical stressors relative to populations naïve to those stressors (Hangartner et al., 2012; Hua et al., 2013; Klerks & Levinton, 1989; Roelofs et al., 2009; Xie & Klerks, 2004). While the fitness (i.e. survival) of both populations was greatest in the uncontaminated reference environment, in the coal ash‐contaminated environment, the resident population had greater fitness than the naïve population, which is consistent with local adaptation to an environmental stressor (Hereford, 2009; Kawecki & Ebert, 2004). Because our study was conducted under natural field conditions, where environmental factors other than the presence of coal ash varied between environments, we cannot definitively isolate the effect of TE tolerance from other effects associated with a contaminated environment. Future laboratory studies isolating the response of organisms to TEs could elucidate these effects and provide insight into the specific mechanisms underlying adaptive responses to TE contaminants, common in human‐impacted environments. We also note that our use of only two populations, where population “type” is not replicated, limits our ability to make strong conclusions regarding the repeatability of our results across other impacted populations. A more robust, landscape‐level approach incorporating more populations in the future will be needed to ascertain the potential for evolution to facilitate local adaptation to habitats impacted by multiple contaminants.

To our knowledge, adaptive responses of amphibians to chemical stressors have only been investigated for a few chemical stressors: pH (Hangartner et al., 2012), pesticides (Hua et al., 2013), and NaCl (Albecker & McCoy, 2017, 2019; Brady, 2012; Gomez‐Mestre & Tejedo, 2003). Each of these studies found populations sourced from environments long impacted by contaminants to be more tolerant to those stressors than naïve populations. However, only two of these studies were conducted under field conditions (Brady, 2012; Gomez‐Mestre & Tejedo, 2003) and one other designed to allow estimation of additive genetic and maternal contributions to the observed phenotypic variation (Hangartner et al., 2012). Our study adds to these results, being the first to document phenotypic divergence between amphibian populations that is consistent with local adaptation to multiple chemical stressors under field conditions.

We saw no evidence that elevated tolerance to coal ash carried a direct fitness cost in the absence of those stressors (i.e. no differences in survival), but it was associated with delays in larval growth and development in the uncontaminated environment. Therefore, elevated tolerance in the ASH population appears to be associated with potentially costly delays in larval development under reference conditions, which may reduce viability of coal ash‐adapted populations in adjacent habitats (Awkerman & Raimondo, 2018), given the negative association between time to metamorphosis and survival (Scott, 1994). Further, given our study only followed individuals up to metamorphosis and did not measure all possible relevant endpoints, there remains the potential for unmeasured fitness costs, including those on fecundity.

### 4.1 Phenotypic plasticity: impact of development in a contaminated environment

Overall, there was little plasticity in size at metamorphosis, such that individuals from both populations emerged at similar sizes, regardless of population, rearing environment, or length of larval development. Larval development was significantly delayed in the presence of coal ash, with no difference in size at metamorphosis, which translated into an overall decrease in growth rate (mg/day). Larval growth and development rates are closely tied to fitness, as quickly developing and growing larvae reduce their risk of mortality associated with predation (Travis et al., 1985) and desiccation as aquatic habitats dry (Travis & Trexler, 1986). In addition, for amphibians, survival to first reproduction is tied to time to and size at the metamorphic transition (Scott, 1994; Smith, 1987). While metamorphosing at larger sizes generally conveys greater probability of survival in terrestrial life stages, optimal size at the transition also depends on conditions in the aquatic environment (Werner, 1986; Wilbur & Collins, 1973). When conditions are optimal for growth and survival, prolonging aquatic development to metamorphose at a larger size will maximize fitness, but if conditions are poor (e.g. high mortality risk or low growth) fitness may be maximized by developing rapidly and metamorphosing at a smaller size (Werner, 1986). Given that exposure to coal ash significantly increases the risk of mortality for developing amphibian larvae, we would predict adaptive plasticity would result in metamorphosis occurring at the minimum size in order to shorten the larval period. Instead, we saw extended larval periods with no change in size at metamorphosis. This suggests that the stressors in the ASH environment reduced growth rates, which translated into delays in reaching the minimum size at which metamorphosis can occur, based on mean sizes at metamorphosis in our study (mass = 0.04 g, SVL = 8.3 mm) being at or below those reported in other studies (mass = 0.06–0.11 g, SVL = 8.6–10.9 mm; Beck & Congdon, 2000; Rumrill et al., 2016; Stark et al., 2015). Delayed development in the ASH environment is in line with previous work suggesting exposure to high levels of the TEs in coal ash can disrupt the function of the hypothalamus‐pituitary‐thyroid axis in amphibians, which is critical to the regulation of metamorphosis (Hopkins, Mendonça et al., 1999).

### 4.2 Population divergence consistent with local adaptation

Fitness in the uncontaminated REF environment was similar for both populations and always greater than fitness in the ASH environment. However, differences in mortality risk between populations in the ASH environment suggest the ASH population is better adapted to those conditions, characterized by multiple chemical stressors. Although we saw no differences in time to metamorphosis or size at metamorphosis between populations when reared in the ASH environment, offspring from the ASH population experienced ~1/3 the risk of mortality in that environment, relative to naïve REF offspring. This observation, where fitness of the population resident in the ASH environment is greater than that of the naïve population, is consistent with a hypothesis that the ASH population has adapted to the elevated levels of trace elements in the ASH environment (Hereford, 2009; Kawecki & Ebert, 2004). We note that while the effects on mortality using linear mixed models considering only whether mortality occurred or not (i.e. not the timing of mortality) were in similar in direction and magnitude as hazard models, they were not statistically significant. While the timing of mortality is less important than whether mortality occurs or not, being able to survive longer does provide evidence that the ASH population is more tolerant to the chemical stressors in the ASH environment. Further, the increased tolerance of the ASH population to coal ash stressors may come with costs as the ASH population developed and grew more slowly than did the REF population when reared in the REF environment. Because larval growth and development can be correlated with fitness, the reduced performance of the ASH population in the absence of contaminants could represent potential fitness costs associated with adaptation to a human‐impacted environment. Fitness‐related costs in the absence of chemical stressors appear to be a common consequence of adaptation to chemical stressors (Agra et al., 2011; Groeters et al., 1994; Shirley & Sibly, 1999; Xie & Klerks, 2004). However, few studies have examined these consequences in populations adapted under field—rather than laboratory selection regimes (Agra et al., 2011; Räsänen et al., 2008). While we did not directly examine the physiological responses of these populations to the two environments here, mechanisms of tolerance to TE stressors in aquatic organisms are well characterized and include upregulation of Na+/K+ ATPases and metallothioneins (Webster & Bury, 2013). Given that genetic assimilation (i.e. constitutive expression) of pathways associated with tolerance to TE stressors has been observed in populations adapted to elevated TEs in the environment (Roelofs et al., 2006, 2009), it is plausible that the TE‐tolerant ASH population has been selected for such constitutive expression (e.g. ATPases, metallothionein). Because metal detoxification is energetically costly, constitutive expression of these pathways would necessarily allocate resources away from growth and development and may partially explain the trade‐offs observed in the absence of CCW stressors.

### 4.3 Quantitative genetic variation in life history traits and their plasticity

Adaptation to anthropogenic stressors requires sufficient additive genetic variation on which selection can act. However, sustained directional or stabilizing selection is also expected to reduce additive genetic variation if the selection pressure is strong enough. Given the constructed ASH environment has been in place for over sixty years (>20 generations assuming conservative estimate of 3 years to first reproduction) and the toxic TEs present there do not degrade, we predicted the sustained contaminant‐induced mortality in that environment would reduce genetic variation in the traits we measured. We found low to moderate heritability for most of the traits examined, and heritability did not differ substantially among population × environment‐specific estimates. Thus, our results suggest the observed adaptive divergence was not accompanied by substantial erosion of additive genetic variation. We did observe differences in environment‐specific heritability estimates for some life history traits, although confidence intervals around estimates overlapped. Specifically, heritability was greater for time to metamorphosis for the ASH population in the REF environment and early growth and size at day 21 for the REF population in the ASH environment. The latter could suggest latent genetic variation revealed by exposure to a novel stressor (i.e. coal ash), consistent with ideas about the role of plasticity in adaptation (Ghalambor et al., 2007, 2015).

Quantitative genetic variation in the plasticity of traits was generally similar to that for environment‐specific trait estimates. While differences in mean trait values may be indicative of past selection, plasticity provides more insight into the potential for coping with current environmental variation. Embryonic and larval mortality were the only traits with estimates of heritability and $h_{\mathrm{pl}}^{2}$ that were not significantly different from zero, which agrees with the expectation that additive genetic variation for traits most closely tied to fitness (i.e. survival) is low compared to other life history traits (Falconer & Mackay, 1996; Roff, 2002). While uncertainty around estimates was large, the heritability estimate for time to metamorphosis for the ASH population in the ASH environment was reduced relative to the REF environment or the REF population in either estimate. These observations could indicate selection in the ASH environment has reduced additive genetic variation in time to metamorphosis, a trait that is impacted by coal ash contamination and thus may be under selection for tolerance. Similarly, $h_{\mathrm{pl}}^{2}$ for this trait was lower in the ASH population relative to REF, which suggests that the ASH population may have a reduced capacity for future evolution of plasticity, relative to the REF population. Generally, there was no strong evidence that past selection in the ASH environment has reduced trait plasticity in the ASH population. Though reaction norms for some traits differed between populations (i.e. early growth and size at day 21), there was little evidence $h_{\mathrm{pl}}^{2}$ was reduced for any traits in the ASH population, with the exception of time to metamorphosis.

Overall, our results suggest that the observed divergence between populations was not accompanied by significant erosion of genetic variation. It is possible that the strength of coal ash‐induced selection may be modest relative to the immigration, mutation, and recombination that maintain genetic variation. Adaptive tolerance to chemical stressors, including TEs found in coal ash, can be associated with few genes of large effect (Macnair, 1991; Shirley & Sibly, 1999) rather than many genes of small effect, as typically assumed by Quantitative genetic theory (Lande, 1982). However, the lack of evidence of genetic erosion is more congruent with selection acting on many genes of small effect.

Our study lacked the statistical power to detect minor differences in additive genetic variation, especially when coupled with the extensive environmental variation inherent to in situ field studies. However, rearing offspring from natural populations in the field provided a unique opportunity to obtain more realistic estimates of heritability and nongenetic maternal effects, as laboratory studies tend to inflate those estimates and reduce phenotypic variation (Falconer & Mackay, 1996). Additive genetic variation is crucial to maintaining the evolutionary potential of populations necessary for response to future environmental change. Thus, identifying reduced survival costs to an anthropogenic selection pressure without reduction in standing genetic variation is of importance for conservation managers seeking to maintain viable populations and understand the consequences of human‐induced environmental change in natural populations.

While there was extensive additive genetic variation for a number of fitness‐related traits, we found very limited evidence of maternal effects. In fact, the only maternal effect estimates not overlapping zero were embryonic survival and size at GS25, with a trend that maternal effects decreased with developmental time and became undetectable by the initiation of the field portion of the study. This pattern is consistent with other studies that have found maternal effects to dominate early in development, but dissipate as development progresses (Cruz & Ibarra, 1997; Montalvo & Shaw, 1994). Further, embryonic survival was the only trait for which maternal effects surpassed genetic effects. In addition to nutrients, hormones, mRNAs, and other molecules (reviewed in Toth, 2015), female amphibians can transfer TEs to their eggs, which can correlate with reduced reproductive success (Metts et al., 2013) and increased malformations (Hopkins et al., 2006). Our results support the notion that nongenetic maternal effects can significantly contribute to offspring survival. However, because the magnitude of maternal effects for both populations was comparable, the bulk of these maternal effects appear to be unrelated to the maternal transfer of contaminants. To our knowledge, only three other studies have investigated the effects of parental exposure to coal ash on the survival and performance of embryonic and larval amphibians (Hopkins et al., 2006; Metts et al., 2012, 2013), and only one of these assessed offspring performance under both reference and coal ash‐contaminated conditions (Metts et al., 2012). In contrast to these previous studies, we did not find evidence of reduced embryonic survival (Hopkins et al., 2006; Metts et al., 2013) or reduced survival to or size at metamorphosis (Metts et al., 2012) in offspring from ASH dams compared to REF dams. However, we did observe similar patterns where offspring derived from the ASH population had delayed development and growth rate relative to the REF population in the absence of coal ash stressors (Metts et al., 2012). Our results suggest that the observed divergence in larval and metamorphic traits between populations reflects genetic divergence rather than differences due to maternal transfer of contaminants.

### 4.4 Conclusions

Our study provides further evidence that natural populations may be able to adapt to human‐impacted environments, characterized by multiple chemical stressors, and that such adaptation could be costly. By incorporating an evolutionary perspective, we found evidence that an amphibian population residing in an anthropogenically degraded habitat has diverged phenotypically from a population in an uncontaminated habitat and that that divergence may come at some cost in uncontaminated environments. The ASH population experienced a reduced risk of mortality in their natal rearing environment impacted by environmental contaminants, compared to the naïve REF population. Importantly, this potentially adaptive tolerance could be costly in the absence of coal ash‐associated stressors, which could negatively impact the average fitness in populations in nearby, uncontaminated wetlands if ASH juveniles disperse to them.

Given the severity and extent of anthropogenically impacted environments is expected to increase in the future, assessing how populations cope with such changes is crucial for understanding the conservation implications of past and continued environmental changes. As a group, amphibians are a globally imperiled taxon, but some species appear able to successfully cope and persist in the face of major environmental perturbations. Our results, coupled with previous studies (e.g. Metts et al., 2012; Rowe et al., 2001), have documented that the unique aquatic environment associated with coal ash disposal sites could act as a selection pressure on *A*. *terrestris*. Specifically, these results suggest that a population has responded adaptively to partially ameliorate fitness reductions associated with this environmental change, but at some cost in other environments. These costs suggest that ultimately the persistence of populations in contaminated environments may depend not only on the potential for local adaptation, but also on the costs of tolerance and the spatial and temporal variation in the presence of contaminants.

## CONFLICTS OF INTEREST

The authors declare no conflict of interest.

## Supporting information

Supplementary Material

## Data Availability

Data will be archived at the University of Georgia's, Savannah River Ecology Laboratory repository, following the acceptance of this paper for publication and available from the corresponding author upon reasonable request.
