# Supplementary material for: Divergence in heritable life history traits suggests potential for local adaptation and trade‐offs associated with a coal ash disposal site
Source: Evol Appl. 2021 Jun 9;14(8):2039–54. doi: 10.1111/eva.13256 (PMC8372081; doi:10.1111/eva.13256)
Supplement: Supplementary file 1 — Supplementary Material [file EVA-14-2039-s001.docx]

**Supplemental Materials**

**Supplemental Figures**


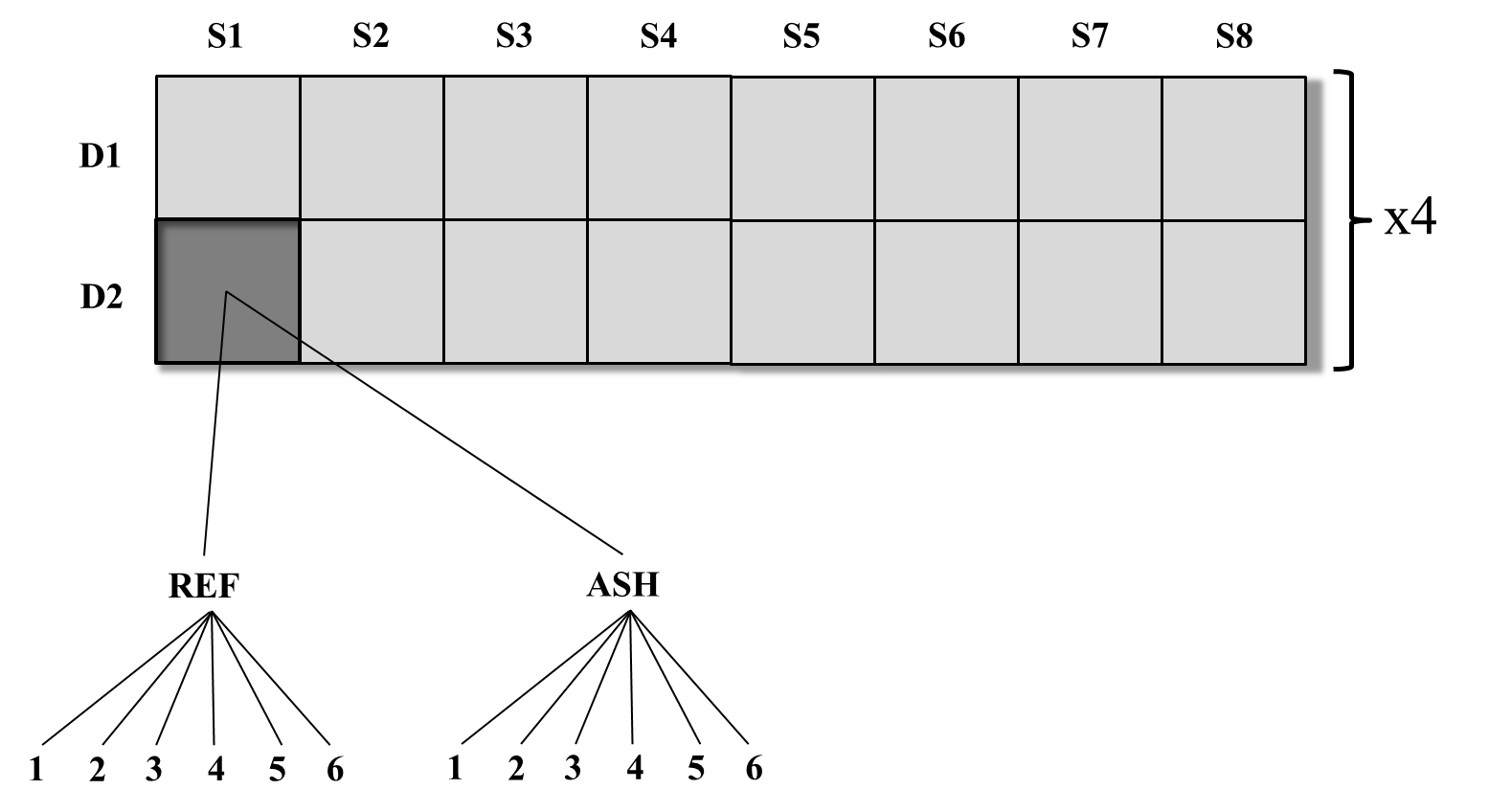


**Figure S1.** Schematic of NC II breeding design used for each population. In each 2 x 8 breeding block, two dams (D) were crossed with eight sires (S). Each square represents a full-sib family. Four of these breeding blocks were used for each population. Six larvae from each full-sib family were reared in each environment (i.e., REF and ASH environments).


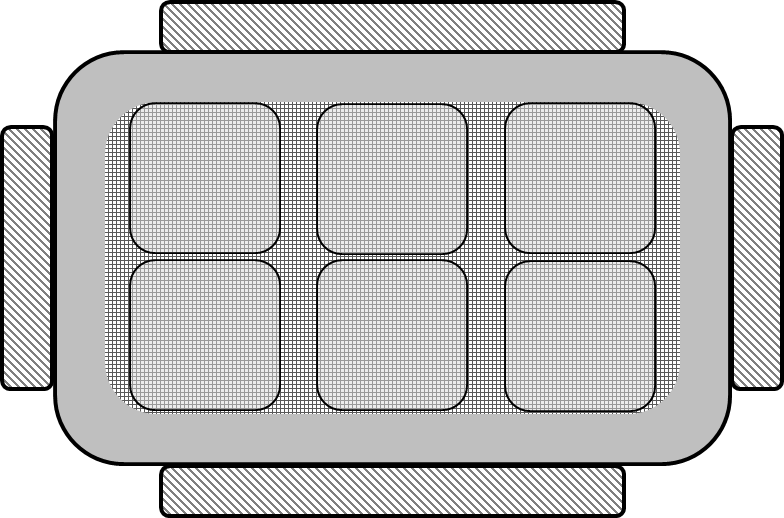


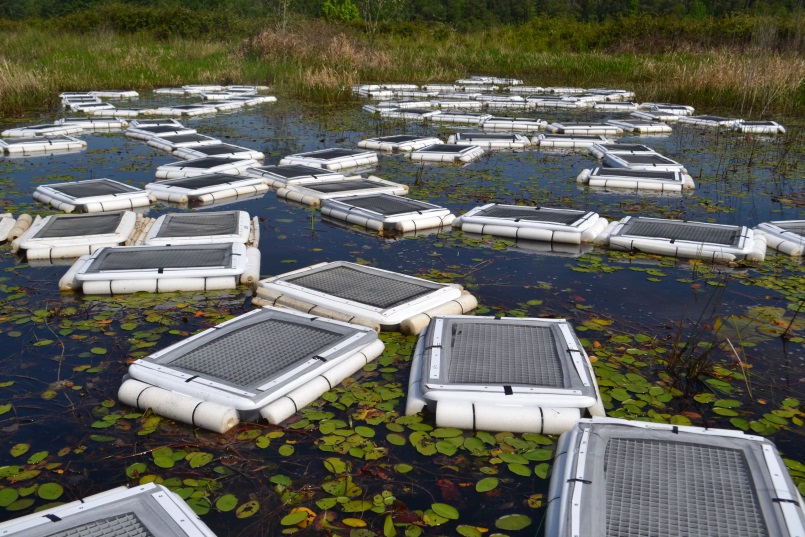


**Figure S2.** Detailed Field Enclosure Design

Standardized foam floatation (diagonal fill) was attached with UV-resistant plastic zip-ties to all four sides of the larger enclosures to maintain a standardized depth of bin and volume of water in the experimental units. The lids and bottoms of both the floating field enclosures and the individual rearing containers within each enclosure (i.e. the six smaller containers seen at top) were removed and fit with non-metallic window screen (crosshatched fill).


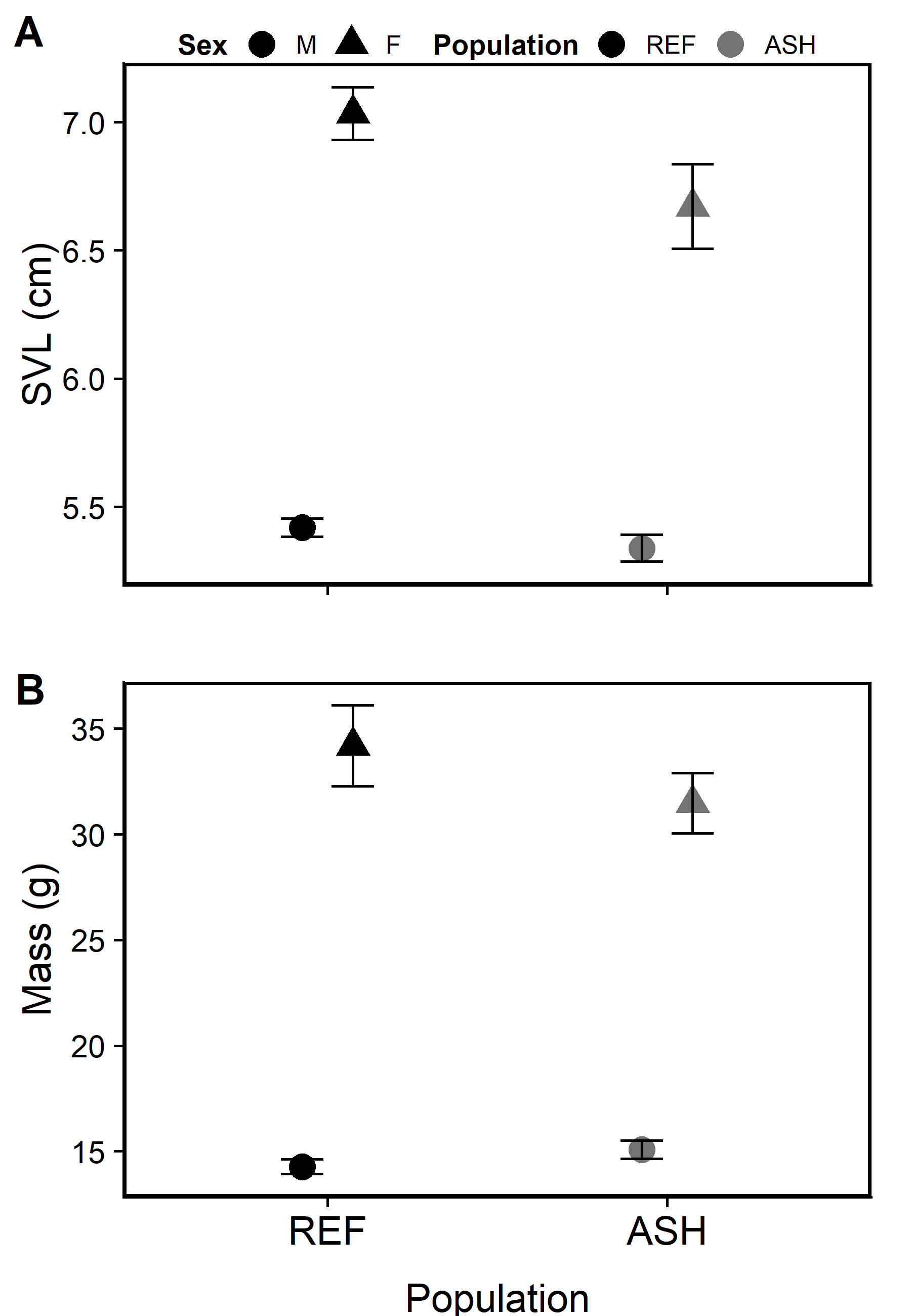


**Figure S3.** Mean (±SE) snout-vent length (A) and body mass (B) for parental toad from REF and ASH sites. There were no significant differences between populations, but both values are consistently greater for females than males.


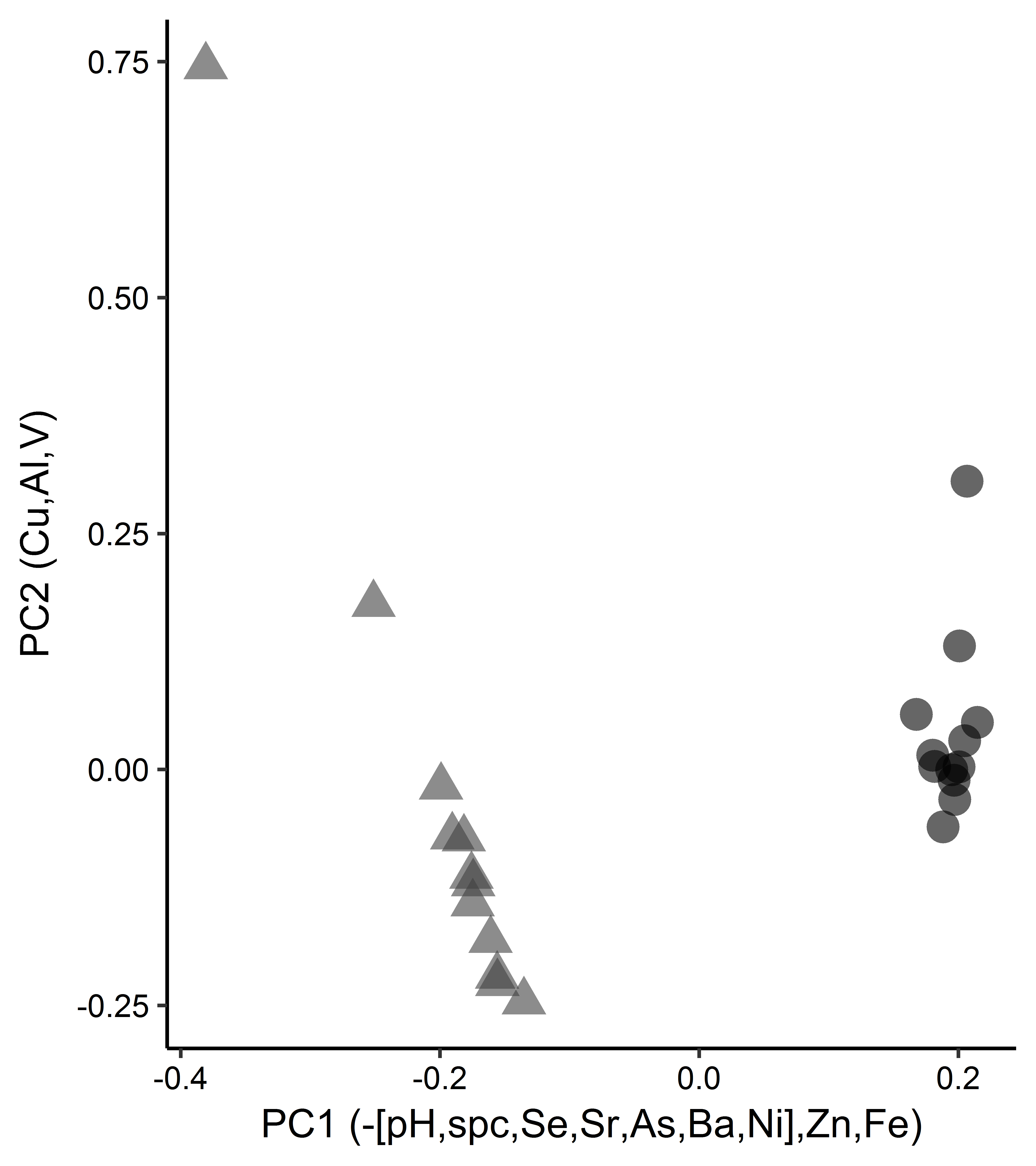


**Figure S4.** Plot of first two, varimax rotated PC-axes associated with chemical parameters of the water at the two sites. PC1 explains 59.1% and PC2 31.7% of the variance in trace element and water quality of the aquatic environments (90.8% total). The two rearing environments (circles = REF, triangles = ASH) separated primarily along the PC1, while PC2 mostly explained within site variation. See supplemental table S2.5 for PC eigenvalues, % variance, and loading scores prior to rotation.


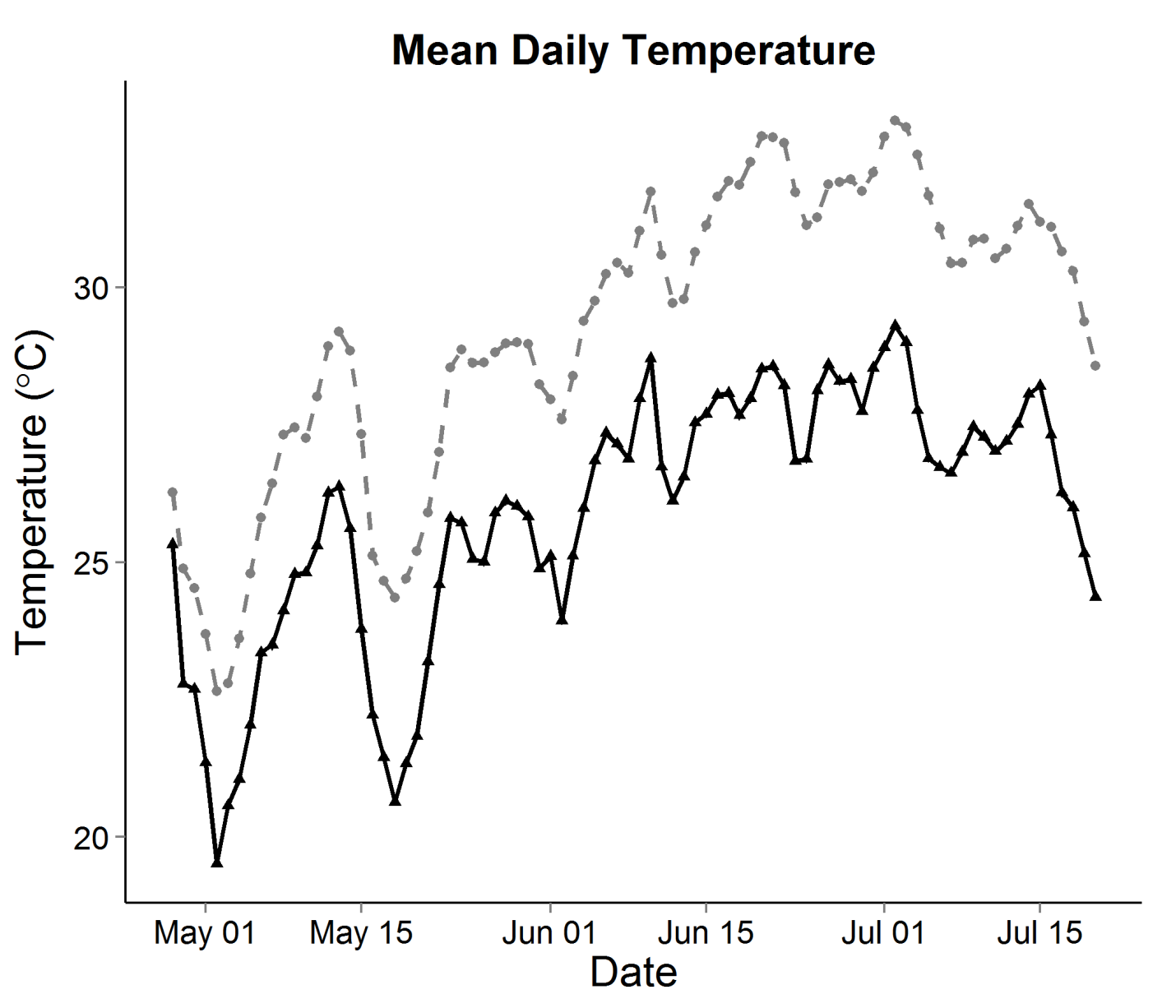


**Figure S5.** Mean daily water temperature profiles across field sites with the REF site in black and the ASH site in gray. The ASH environment was consistently warmer and had less thermal variability.


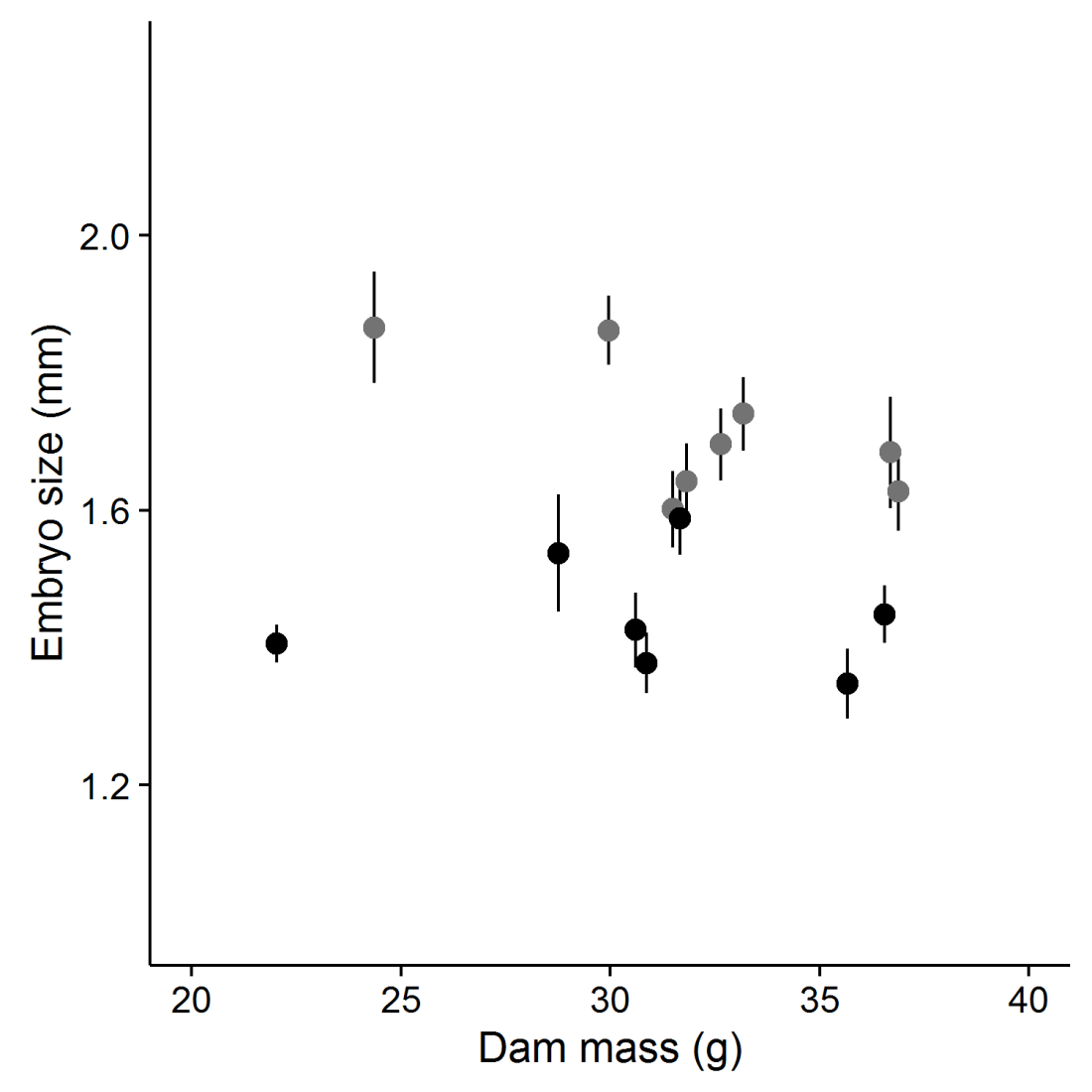


**Figure S6.** Plot of mean embryo size (95% CI) against dam mass. While there was no relationship between dam size and embryo size in the REF population (black), there was a slight negative relationship between the two in the ASH population, counter to other research examining this relationship in amphibians (Laugen, Laurila, & Merilä, 2002). Laugen et al. (2002) found a positive relationship only in some populations, however, in others there was no discernable relationship between the two.

**
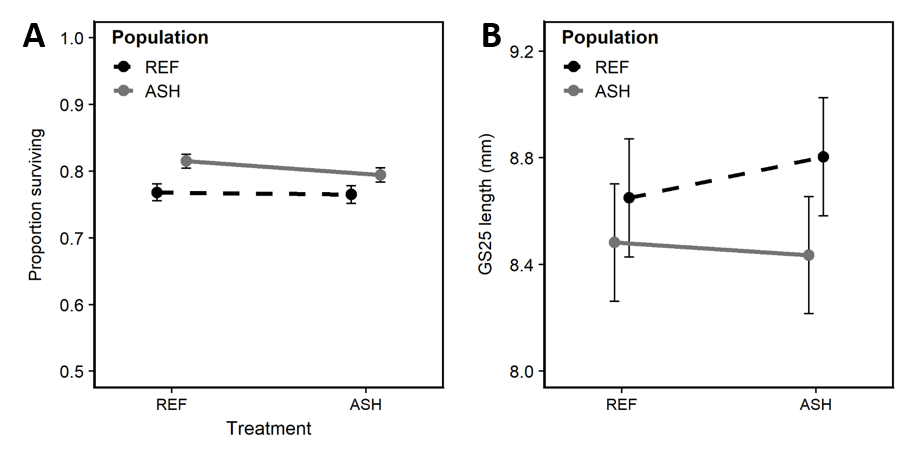
**

**Figure S7.** Means and errors associated with A) survival through embryonic development and B) size (total length) at the free-swimming stage (i.e., Gosner stage 25).

**
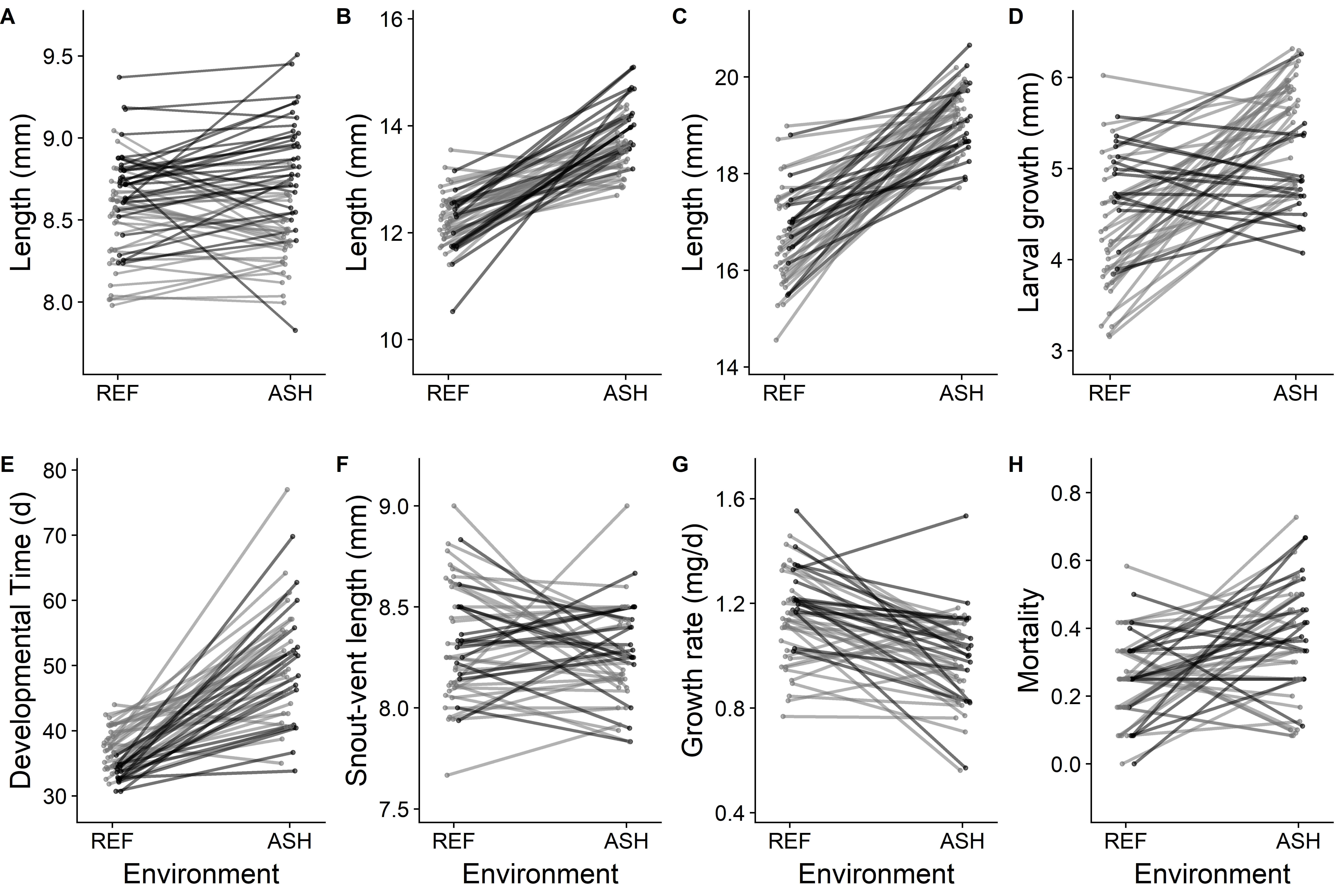
**

**Figure S8.** Sire specific reaction norms in each population (REF = black, ASH = gray) for size at GS25 (A), larval size at days 7 and 21 (B,C), early larval growth (D), time to and size at metamorphosis (E,F), mean daily growth rate (G), and larval mortality (H).

**Supplemental Tables**

**Table S1.** Summary of water quality and major element concentrations in aquatic environment at study sites. Values significantly greater in the ASH environment are indicated in bold (p ≤ 0.05).

**Table S2.** Summary of trace element concentrations (μg/L) in aquatic environment at study sites. Bolded values are those that are significantly greater in that environment (p ≤ 0.05). BDL = below instrument detection limit.

**Table S3.** Table summarizing concentrations of select trace elements in adult toad livers from the reference (REF) and CCW-disposal site (ASH) populations used to generate offspring for our study. Bolded values are those that are significantly greater in that environment (p ≤ 0.05).

| pop | element | n | mean | median | sd | se | min | max |
| --- | --- | --- | --- | --- | --- | --- | --- | --- |
| REF | As | 36 | 0.79 | 0.71 | 0.38 | 0.06 | 0.26 | 1.64 |
| **ASH** | **As** | **34** | **2.37** | **2.02** | **1.50** | **0.26** | **0.95** | **9.23** |
| REF | Cd | 36 | 0.50 | 0.30 | 0.56 | 0.09 | 0.09 | 3.23 |
| ASH | Cd | 34 | 0.48 | 0.43 | 0.26 | 0.04 | 0.20 | 1.21 |
| REF | Cu | 36 | 216.72 | 190.71 | 171.68 | 28.61 | 15.28 | 814.40 |
| **ASH** | **Cu** | **34** | **388.87** | **273.18** | **323.20** | **55.43** | **40.63** | **1273.37** |
| REF | Se | 36 | 5.70 | 4.82 | 2.80 | 0.47 | 1.82 | 14.73 |
| **ASH** | **Se** | **34** | **29.38** | **19.07** | **29.35** | **5.03** | **7.09** | **150.09** |
| REF | Sr | 35 | 0.33 | 0.25 | 0.18 | 0.03 | 0.13 | 0.86 |
| **ASH** | **Sr** | **35** | **1.05** | **0.89** | **0.63** | **0.11** | **0.18** | **3.48** |
| REF | V | 36 | 1.51 | 0.93 | 1.45 | 0.24 | 0.30 | 5.11 |
| ASH | V | 34 | 1.90 | 1.43 | 1.28 | 0.22 | 0.80 | 5.41 |
| REF | Zn | 36 | 83.97 | 82.66 | 14.95 | 2.49 | 48.89 | 139.82 |
| ASH | Zn | 35 | 89.52 | 89.52 | 20.94 | 3.54 | 0.40 | 120.08 |

**Table S4.** Table summarizing clutch x treatment combinations from the (A) embryonic and (B) larval studies that did not have full set of replicates (i.e., embryonic < 3 and larval < 6). The ‘*n*’ column shows number of experimental units (A) or individuals (B) for given clutch x treatment combinations that were included in the study.

A

B

**Table S5.** Major cation and trace element levels in water samples collected from both REF and ASH environments used in laboratory embryonic study. Samples were taken from each carboy, containing field collected water, used to fill experimental units at the start of the trial (start) and a random subset of experimental units at the end of the trial (final). All elemental values are reported in μg/L. BDL = values below the instrument detection limit.

**Table S5 (continued)**

**Table S6.** Water quality, major cation, and trace element levels at sampling locations (spatial blocks) within ASH and REF field sites. On each sampling date, two measurements were made at each spatial block, one in a random experimental enclosure (BIN) and one outside the enclosure. All element values reported in μg/L. spc = specific conductance reported in μS/cm.

**Table S6 (continued)**

**Table S7.** Principal component loadings of water quality and trace element levels in environmental water samples taken in field. Specific conductance (spc) was modeled in µS/cm and trace element concentrations as ug/L. The first two PCs sufficiently explained the environmental variation in the data.

**Table S8.** Quantitative genetic variance component means (HPDI) derived from population and environment-specific models (Supplement S3.3). CV_A_ was calculated as $({\sqrt{V_{A}}}/\bar{X}) \times100$, where $\bar{X}$ equals the population x environment-specific trait mean. I_A_ is a measure of evolvability and calculated as $V_{A}/\bar{X}^{2}$.

|  |  |  |  | **size GS25** |  |  |  |
| --- | --- | --- | --- | --- | --- | --- | --- |
| pop | env | VA | VM | VD | VP | CVA | IA |
| REF | REF | 0.0009 (0.0005-0.0018) | 0.0004 (0.0000-0.0019) | 0.0008 (0.0004-0.0014) | 0.0017 (0.0012-0.0032) | 3.2234 (2.3068-4.4861) | 0.1070 (0.0532-0.2013) |
|  | ASH | 0.0009 (0.0004-0.0016) | 0.0007 (0.0000-0.0026) | 0.0007 (0.0004-0.0012) | 0.0019 (0.0012-0.0038) | 3.0801 (2.1809-4.3270) | 0.0978 (0.0476-0.1872) |
| ASH | REF | 0.0006 (0.0003-0.0012) | 0.0006 (0.0000-0.0023) | 0.0005 (0.0002-0.0008) | 0.0016 (0.0010-0.0032) | 2.7016 (1.9938-3.6938) | 0.0748 (0.0398-0.1364) |
|  | ASH | 0.0007 (0.0004-0.0012) | 0.0006 (0.0000-0.0021) | 0.0006 (0.0003-0.0010) | 0.0017 (0.0012-0.0032) | 2.7731 (2.0385-3.7935) | 0.0789 (0.0416-0.1439) |
|  |  |  |  |  |  |  |  |
|  |  |  |  | **size 1** |  |  |  |
| pop | env | VA | VM | VD | VP | CVA | IA |
| REF | REF | 0.002 (0.0007-0.0049) | 0.0028 (0.0000-0.0163) | 0.0014 (0.0006-0.0031) | 0.0079 (0.0036-0.023) | 4.0406 (2.4686-6.5255) | 0.1743 (0.0609-0.4258) |
|  | ASH | 0.0021 (0.0007-0.0051) | 0.0022 (0.0000-0.0118) | 0.0019 (0.0007-0.0041) | 0.0068 (0.0033-0.0175) | 3.8504 (2.3612-6.2616) | 0.1820 (0.0558-0.3924) |
| ASH | REF | 0.0012 (0.0005-0.0023) | 0.0005 (0.0000-0.0022) | 0.0010 (0.0005-0.0019) | 0.0042 (0.0030-0.0074) | 3.0764 (2.1319-4.3926) | 0.0981 (0.0454-0.1930) |
|  | ASH | 0.0011 (0.0005-0.0021) | 0.0003 (0.0000-0.0016) | 0.0010 (0.0005-0.0018) | 0.0040 (0.0027-0.0075) | 2.8967 (1.9995-4.1070) | 0.0868 (0.0400-0.1687) |
|  |  |  |  |  |  |  |  |
|  |  |  |  |  |  |  |  |
|  |  |  |  |  |  |  |  |
|  |  |  |  | **size 2** |  |  |  |
| pop | env | VA | VM | VD | VP | CVA | IA |
| REF | REF | 0.0024 (0.0008-0.0062) | 0.0036 (0.0000-0.0151) | 0.0028 (0.0010-0.0063) | 0.0116 (0.0053-0.0263) | 3.8754 (2.2801-6.4548) | 0.1619 (0.0520-0.4166) |
|  | ASH | 0.0026 (0.0037-0.0183) | 0.0019 (0.000-0.0125) | 0.0034 (0.0012-0.0074) | 0.0070 (0.0037-0.0183) | 3.8511 (2.2557-6.4355) | 0.1602 (0.0509-0.4142) |
| ASH | REF | 0.0020 (0.0008-0.0042) | 0.0002 (0.0000-0.0018) | 0.0020 (0.0008-0.0040) | 0.0070 (0.0047-0.0133) | 3.5869 (2.3200-5.3058) | 0.1345 (0.0538-0.2815) |
|  | ASH | 0.0011 (0.005-0.0021) | 0.0004 (0.0000-0.0016) | 0.0010 (0.0005-0.0019) | 0.0042 (0.0028-0.0078) | 2.5136 (1.7464-3.5739) | 0.0654 (0.0305-0.1277) |
|  |  |  |  |  |  |  |  |
|  |  |  |  | **early growth** |  |  |  |
| pop | env | VA | VM | VD | VP | CVA | IA |
| REF | REF | 0.0073 (0.0013-0.0244) | 0.0057 (0.0000-0.0347) | 0.0152 (0.0021-0.0464) | 0.0596 (0.0397-0.1104) | 12.4545 (5.5007-24.9190) | 1.8650 (0.3026-6.2252) |
|  | ASH | 0.0089 (0.0014-0.0300) | 0.0045 (0.0052-0.0296) | 0.0188 (0.0023-0.0548) | 0.0399 (0.0269-0.0690) | 13.4994 (5.7346-27.3732) | 2.1519 (0.3289-7.4929) |
| ASH | REF | 0.0109 (0.0016-0.0356) | 0.0104 (0.0000-0.0420) | 0.0192 (0.0022-0.0570) | 0.1163 (0.0878-0.1778) | 17.3540 (6.8622-35.2074) | 3.8832 (0.4710-12.3985) |
|  | ASH | 0.0029 (0.0009-0.0070) | 0.0009 (0.0000-0.0051) | 0.0033 (0.0010-0.0080) | 0.0214 (0.0169-0.0303) | 7.1022 (4.1098-11.4914) | 0.5417 (0.1689-1.3205) |
|  |  |  |  |  |  |  |  |
|  |  |  |  |  |  |  |  |
|  |  |  |  |  |  |  |  |
|  |  |  |  | **ttm** |  |  |  |
| pop | env | VA | VM | VD | VP | CVA | IA |
| REF | REF | 0.0024 (0.0008-0.0060) | 0.0021 (0.0000-0.0126) | 0.0023 (0.0008-0.0052) | 0.0096 (0.0052-0.0229) | 3.0866 (1.8297-5.1225) | 0.1025 (0.0335-0.2624) |
|  | ASH | 0.0172 (0.0020-0.0586) | 0.0088 (0.0000-0.0549) | 0.0147 (0.0019-0.0497) | 0.0715 (0.0371-0.1637) | 7.2395 (2.7089-14.5959) | 0.6245 (0.0734-2.1304) |
| ASH | REF | 0.0028 (0.0010-0.0064) | 0.0003 (0.0000-0.0026) | 0.0028 (0.0010-0.0059) | 0.0096 (0.0070-0.0156) | 3.3166 (1.9935-5.1128) | 0.1164 (0.0397-0.2614) |
|  | ASH | 0.0054 (0.0012-0.0150) | 0.0010 (0.0000-0.0072) | 0.0086 (0.0016-0.0231) | 0.0524 (0.0352-0.1016) | 4.1788 (2.1133-7.2987) | 0.1936 (0.0447-0.5327) |
|  |  |  |  |  |  |  |  |
|  |  |  |  | **svl** |  |  |  |
| pop | env | VA | VM | VD | VP | CVA | IA |
| REF | REF | 0.0016 (0.0006-0.0038) | 0.0019 (0.0000-0.0102) | 0.0012 (0.0005-0.0025) | 0.0047 (0.0020-0.0140) | 4.2141 (2.678-6.6997) | 0.1885 (0.0717-0.4489) |
|  | ASH | 0.0016 (0.0006-0.0039) | 0.0019 (0.0000-0.0116) | 0.0014 (0.0006-0.0029) | 0.0049 (0.0021-0.0153) | 4.2819 (2.6848-6.852) | 0.1946 (0.0721-0.4695) |
| ASH | REF | 0.0011 (0.0005-0.0021) | 0.0004 (0.0000-0.0016) | 0.0011 (0.0005-0.0019) | 0.0033 (0.0022-0.0064) | 3.5076 (2.4323-5.0152) | 0.1273 (0.0592-0.2515) |
|  | ASH | 0.0010 (0.0005-0.0020) | 0.0003 (0.0000-0.0013) | 0.0013 (0.0007-0.0023) | 0.0027 (0.0018-0.0051) | 3.4305 (2.3858-4.8943) | 0.1217 (0.0569-0.2395) |
|  |  |  |  |  |  |  |  |
|  |  |  |  |  |  |  |  |
|  |  |  |  |  |  |  |  |
|  |  |  |  | **mass** |  |  |  |
| pop | env | VA | VM | VD | VP | CVA | IA |
| REF | REF | 0.0042 (0.0011-0.0114) | 0.0018 (0.0000-0.0149) | 0.0044 (0.0012-0.0107) | 0.0123 (0.0074-0.0273) | 3.9100 (2.106-6.6900) | 0.1671 (0.0444-0.4476) |
|  | ASH | 0.0037 (0.0010-0.0106) | 0.0027 (0.0000-0.0169) | 0.003 (0.0009-0.0077) | 0.0128 (0.0071-0.0297) | 3.6295 (1.9535-6.4181) | 0.1450 (0.0382-0.4119) |
| ASH | REF | 0.0031 (0.0010-0.0073) | 0.0006 (0.0000-0.0040) | 0.0039 (0.0013-0.0084) | 0.0133 (0.0094-0.0225) | 3.3925 (2.0151-5.3977) | 0.1227 (0.0406-0.2914) |
|  | ASH | 0.0026 (0.0009-0.0061) | 0.0003 (0.0000-0.0024) | 0.0035 (0.0012-0.0077) | 0.0088 (0.0067-0.0126) | 3.1131 (1.8666-4.8843) | 0.1030 (0.0348-0.2386) |
|  |  |  |  |  |  |  |  |
|  |  |  |  | **growth rate (mg/d)** |  |  |  |
| pop | env | VA | VM | VD | VP | CVA | IA |
| REF | REF | 0.0057 (0.0012-0.0171) | 0.0020 (0.0000-0.0154) | 0.0073 (0.0016-0.0194) | 0.0225 (0.0148-0.0416) | NA | NA |
|  | ASH | 0.0154 (0.0019-0.0529) | 0.0052 (0.0000-0.0422) | 0.0149 (0.0018-0.0509) | 0.06300 (0.0359-0.1414) | NA | NA |
| ASH | REF | 0.0070 (0.0016-0.0182) | 0.0000 (0.0000-0.0042) | 0.0103 (0.0024-0.0236) | 0.0263 (0.0204-0.0381) | NA | NA |
|  | ASH | 0.0049 (0.0011-0.0142) | 0.0007 (0.0000-0.0060) | 0.0083 (0.0016-0.0234) | 0.0620 (0.0418-0.1186) | NA | NA |
|  |  |  |  |  |  |  |  |
|  |  |  |  | **probability of mortality** |  |  |  |
| pop | env | VA | VM | VD | VP | CVA | IA |
| REF | REF | 0.3602 (0.0004-1.6691) | 0.2889 (0.0000-1.9845) | 0.3138 (0.0003-1.4389) | 1.7325 (1.0523-3.9680) | NA | NA |
|  | ASH | 0.2687 (0.0004-1.2915) | 0.2937 (0.0000-1.8203) | 0.4175 (0.0007-1.8251) | 2.0123 (1.1160-5.0534) | NA | NA |
| ASH | REF | 0.1084 (0.0001-0.5154) | 0.0434 (0.0000-0.3446) | 0.4172 (0.0017-1.3487) | 1.3476 (1.0627-2.0872) | NA | NA |
|  | ASH | 0.3220 (0.0009-1.1359) | 0.0637 (0.0000-0.5311) | 0.2287 (0.0003-0.9653) | 1.6360 (1.1630-2.9991) | NA | NA |

**Supplemental Text**

**Text S1. Breeding adult size and trace element body burdens**

We tested for differences in adult sizes and body burdens of TEs (As, Ba, Cd, Cu, Se, Sr, V, Zn) between populations using MANOVA, with population of origin and sex as fixed effects using type III sums of squares. The interaction between population and sex was not significant (p = 0.8063), so it was not included in the final models. Body condition indices (BCI) for each adult were derived by taking residuals from models regressing SVL on mass (Schulte-Hostedde, Zinner, Millar, & Hickling, 2005). These residuals were then used as the response variables in a simple linear regression including population of origin and sex as fixed effects.

*Adult size and body burden*

Adults from the two populations did not differ significantly in terms of SVL (F_1,71_ = 1.38, p = 0.244) or mass (F_1,71_ = 1.42, p = 0.238). However, both SVL and mass were consistently larger for females in both populations (Figure S3).

We found adults from the ASH population accumulated higher levels of trace elements (i.e., liver concentrations) than those from REF (MANOVA: F_1,68_ = 12.31, p < 0.001) and that overall, females tended to accumulate higher levels of elements than males (MANOVA: F_1,68_ = 7.11, p < 0.001). Univariate models showed that body burdens of ASH adults were greater than (As, Cu, Se, and Sr) or not different (Cd, V, Zn) from REF adults (Table S3).

**Text S2. *Artificial Fertilization Methods***

Females were injected with 250 IU of human chorionic gonadotropin to induce ovulation and placed individually in 6 qt plastic shoeboxes containing simplified amphibian Ringer’s solution (ASH: 0730h on 4/15/14; REF: 2400h on night of 4/17/14 - 4/18/14). Females began laying eggs 10-15 h post-injection. As eggs became available, we euthanized males in 3% MS-222, rinsed them with water and removed their testes. Each testis was placed into a separate 1.5 mL microcentrifuge tube containing 500 μL of reconstituted soft water (US Environmental Protection Agency, 2002) and macerated before diluting with an additional 1.0 mL of soft water resulting in a sperm suspension. To achieve our desired breeding design, we cut strings of eggs from each female into sections of approximately equal length and distributed them among eight 60 mL plastic weigh cups (i.e., one cup for each sire x dam combination). Then, to fertilize eggs, we rinsed eggs in soft water to activate them before adding 600 μL of the sperm suspensions pooled from both testis onto the eggs of each of the two females in the breeding block. After 15 min, the weigh cups containing the eggs and sperm were flooded with soft water and left overnight. Following fertilization, female toads were also euthanized, and males and females were frozen.

**Text S3:**

*Population x environment interaction models*

*Embryonic survival*

library(MCMCglmm)

# parameter expanded prior for multinomial MCMCglmm model

prior3.1=list(

R = list (V = 1, fix = 1

G = list (G1 = list(V = 1, nu = 1, alpha.mu = 0, alpha.V = 1000),

G2 = list(V = 1, nu = 1, alpha.mu = 0, alpha.V = 1000),

G3 = list(V = 1, nu = 1, alpha.mu = 0, alpha.V = 1000),

G4 = list(V = 1, nu = 1, alpha.mu = 0, alpha.V = 1000)))

# multinomial mixed effect model for embryonic survival

mpr <- MCMCglmm(cbind(surv, dead) ~ 1 + initial + pop:embryo.size + pop

+ site + pop:site ,

random = ~ pop:sire + pop:dam + pop:sire:dam + ID,

family = "multinomial2",

prior = prior3.1,

data = ante2014.embryo.survival,

nitt = 3000000,

burnin = 100000,

thin = 1000,

slice = T,

verbose = FALSE)

# visual and numerical inspection of model for autocorrelation and chain mixing

plot(mpr$VCV)

plot(mpr$Sol)

autocorr.diag(mpr$Sol) # should be < 0.1 for first lag

autocorr.diag(mpr$VCV) # should be < 0.1 for first lag

effectiveSize(mpr$Sol)

effectiveSize(mpr$VCV)

# Obtaining fixed effect estimates and their statistical significance

summary(mpr)

*Larval and metamorphic life history traits*

library(lme4)

library(car)

trait.full.mod <- lmer(trait ~ pop + site + pop:site + (1|pop:sire) +

(1|pop:dam) + (1 | pop:sire:dam) +

(1|site:loc),

na.action = na.omit, REML = T, data=ante2014)

summary(trait.full.mod) # obtain fixed effect term estimates

Anova(trait.full.mod, type= "III") # statistical significance of fixed effects with Type-3 error

*Within population plasticity models*

*Embryonic survival*

library(MCMCglmm)

prior.embryo.1 = list(

R = list (V = 1, fix = 1),

G = list (G1 = list(V = 1, nu = 1, alpha.mu = 0, alpha.V = 1000),

G2 = list(V = 1, nu = 1, alpha.mu = 0, alpha.V = 1000),

G3 = list(V = 1, nu = 1, alpha.mu = 0, alpha.V = 1000),

G4 = list(V = 1, nu = 1, alpha.mu = 0, alpha.V = 1000),

G5 = list(V = 1, nu = 1, alpha.mu = 0, alpha.V = 1000),

G6 = list(V = 1, nu = 1, alpha.mu = 0, alpha.V = 1000)))

#multinomial generalized linear mixed effect model for embryonic survival

mpr <- MCMCglmm(cbind(surv, (dead)) ~ 1 + site ,

random = ~ sire + dam + sire:dam + site:sire + site:dam +

site:sire:dam ,

family = "multinomial2",

prior = prior.embryo.1,

data = ante2014.embryo,

nitt = 3000000,

burnin = 100000,

thin = 1000,

slice = T,

verbose = FALSE)

*Larval and metamorphic traits*

prior3.1=list(

R = list (V = 1, nu = 0.002),

G = list (G1 = list(V = 1, nu = 0.002),

G2 = list(V = 1, nu = 0.002),

G3 = list(V = 1, nu = 0.002),

G4 = list(V = 1, nu = 0.002),

G5 = list(V = 1, nu = 0.002),

G6 = list(V = 1, nu = 0.002),

G7 = list(V = 1, nu = 0.002)))

# linear mixed effect model for larval and metamorphic traits

mpr1 <- MCMCglmm(trait.x ~ 1 + site ,

random = ~ sire + dam + sire:dam + site:loc + site:sire +

site:dam + site:sire:dam ,

family = "gaussian",

prior = prior3.1,

data = ante2014QG1,

nitt = 2020000,

burnin = 100000,

thin = 1000,

DIC = TRUE,

verbose = FALSE)

*Probability of metamorphosis*

prior.meta.prob = list(

R = list (V = 1, fix = 1),

G = list (G1 = list(V = 1, nu = 1, alpha.mu = 0, alpha.V = 1000),

G2 = list(V = 1, nu = 1, alpha.mu = 0, alpha.V = 1000),

G3 = list(V = 1, nu = 1, alpha.mu = 0, alpha.V = 1000),

G4 = list(V = 1, nu = 1, alpha.mu = 0, alpha.V = 1000),

G5 = list(V = 1, nu = 1, alpha.mu = 0, alpha.V = 1000),

G6 = list(V = 1, nu = 1, alpha.mu = 0, alpha.V = 1000),

G7 = list(V = 1, nu = 1, alpha.mu = 0, alpha.V = 1000)))

mpr1 <- MCMCglmm(meta ~ 1 + site , # meta = 0/1

random = ~ sire + dam + sire:dam + site:loc + site:sire +

site:dam + site:sire:dam,

family = "threshold",

prior = prior.meta.prob,

data = ante2014,

nitt = 2000000,

burnin = 10000,

thin = 1000,

slice = T,

DIC = TRUE,

verbose = FALSE)

*Population and environment specific quantitative genetic models*

*Embryonic survival*

prior.embryo.surv=list(

R = list (V = 1, fix = 1),

G = list (G1 = list(V = 1, nu = 1, alpha.mu = 0, alpha.V = 1000),

G2 = list(V = 1, nu = 1, alpha.mu = 0, alpha.V = 1000),

G3 = list(V = 1, nu = 1, alpha.mu = 0, alpha.V = 1000),

G4 = list(V = 1, nu = 1, alpha.mu = 0, alpha.V = 1000)))

mpr <- MCMCglmm(cbind(surv, dead) ~ 1 + initial ,

random = ~ sire + dam + sire:dam + ID ,

family = "multinomial2",

prior = prior3.4,

data = ante2014QG1,

nitt = 10000000,

burnin = 500000,

thin = 2000,

slice = T,

verbose = FALSE)

*Larval and metamorphic traits*

prior.trait = list(

R = list (V = 1, nu = 0.002),

G = list (G1 = list(V = 1, nu = 0.002),

G2 = list(V = 1, nu = 0.002),

G3 = list(V = 1, nu = 0.002),

G4 = list(V = 1, nu = 0.002)))

mpr1 <- MCMCglmm(trait.x ~ 1,

random = ~sire + dam + sire:dam + loc,

family = "gaussian",

prior = prior.trait,

data = ante2014QG1,

nitt = 1000000,

burnin = 100,

thin = 100,

DIC = TRUE,

verbose = FALSE)

*Probability of metamorphosis*

prior.meta.prob = list(

R = list (V = 1, fix = 1),

G = list (G1 = list(V = 1, nu = 1, alpha.mu = 0, alpha.V = 1000),

G2 = list(V = 1, nu = 1, alpha.mu = 0, alpha.V = 1000),

G3 = list(V = 1, nu = 1, alpha.mu = 0, alpha.V = 1000),

G4 = list(V = 1, nu = 1, alpha.mu = 0, alpha.V = 1000)))

mpr <- MCMCglmm(meta ~ 1 ,

random = ~sire + dam + sire:dam + loc ,

family = "threshold",

prior = prior.meta.prob,

data = ante2014QG1,

nitt = 4000000,

burnin = 1000,

thin = 500,

DIC = TRUE,

slice = T,

verbose = FALSE)
